# Oxidized analogs of Di(1*H*-indol-3-yl)methyl-4-substituted benzenes are NR4A1-dependent UPR inducers with potent and safe anti-cancer activity

**DOI:** 10.18632/oncotarget.25285

**Published:** 2018-05-18

**Authors:** Marisa Sanchez, Zebin Xia, Elizabeth Rico-Bautista, Xihua Cao, Michael Cuddy, David J. Castro, Ricardo G. Correa, Liqun Chen, Jinghua Yu, Andrey Bobkov, Vivian Ruvolo, Michael Andreeff, Robert G. Oshima, Shu-Ichi Matsuzawa, John C. Reed, Xiao-Kun Zhang, Donna Hansel, Dieter A. Wolf, Marcia I. Dawson

**Affiliations:** ^1^ Cancer Center, Sanford Burnham Prebys Medical Discovery Institute, La Jolla, USA; ^2^ School of Pharmaceutical Sciences, Fujian Provincial Key Laboratory of Innovative Drug Target Research and Center for Stress Signaling Networks, Xiamen University, Xiamen, China; ^3^ Oregon Health and Science University School of Medicine, Portland, OR, USA; ^4^ Section of Molecular Hematology and Therapy, Department of Stem Cell Transplantation and Cellular Therapy, Department of Leukemia, The University of Texas M.D. Anderson Cancer Center, Houston, USA; ^5^ Present address: Department of Neurology, Kyoto University Graduate School of Medicine, Kyoto, Japan; ^6^ Present address: Roche, Pharma Research and Early Development, Basel, Switzerland; ^7^ Department of Pathology, University of California San Diego, San Diego, CA, USA

**Keywords:** orphan nuclear receptor 4A1, unfolded protein response, apoptosis, prostate cancer, oxidation products

## Abstract

Di(1*H*-indol-3-yl)(4-trifluoromethylphenyl)methane (DIM-Ph-4-CF_3_) is an analog of orphan nuclear receptor 4A1 (NR4A1) ligand cytosporone B. We have synthesized several oxidation products of DIM-Ph-4-CF_3_, focusing on analogs with electron-withdrawing or donating groups at their phenyl ring 4-positions, and examined their anti-cancer activity and mechanism-of-action. Mesylates (DIM-Ph-4-X^+^ OMs^–^s) having CF_3_, CO_2_Me and Cl groups were more effective inhibitors of cancer cell viability than their precursors. ^19^F NMR spectroscopy and differential scanning calorimetry strongly indicated interactions of DIM-Ph-4-CF_3_^+^ OMs^–^ with the NR4A1 ligand binding domain, and compound-induced apoptosis of prostate cancer cells was dependent on NR4A1. DIM-Ph-4-CF3^+^ OMs^–^ showed robust inhibition of LNCaP prostate cancer xenografts with no apparent toxicity. *In vitro* and *in vivo*, DIM-Ph-4-CF3^+^ OMs^–^ activated proapoptotic unfolded protein response (UPR) signaling in prostate cancer cells. Independently of DIM-Ph-4-CF_3_^+^ OMs^–^, the bulk of NR4A1 localized to the cytoplasm in various cancer cell lines, suggesting a cytoplasmic mechanism-of-action of DIM-Ph-4-CF_3_^+^ OMs^–^ in UPR induction and cell death. In summary, the data suggest that oxidized analogs of DIM-Ph-4-CF3 possess potent and safe anti-cancer activity which is mediated through UPR signaling downstream of NR4A1 binding.

## INTRODUCTION

Orphan nuclear receptor 4A1 (NR4A1; human TR3, mouse Nur77) plays roles in regulating cancer cell viability. NR4A1 expression or over-expression is detected in cancer cell lines and tumors and is higher in prostate cancer biopsy specimens than adjacent normal tissue [1]. Depending on cell type, NR4A1 stimulates cell-cycle progression and proliferation [2–5] or cell-cycle arrest and death [5–9]. In the nucleus, NR4A1 functions as a transcription factor (TF) by binding to NR4A1-regulated gene promoters to stimulate proliferation [3, 4]. In the cytoplasm NR4A1 signaling is nongenomic and pro-apoptotic through interaction with other proteins [7, 9]. Apoptotic agents can induce nongenomic activity involving NR4A1 over-expression, nuclear export into the cytoplasm and interaction with mitochondrial membrane-bound Bcl-2 leading to Bcl-2 conformational change and apoptosis [6, 7, 9]. NR4A1 also translocates to the endoplasmic reticulum (ER) during ER stress-induced apoptosis [10, 11] leading to loss of ER Ca(II) homeostasis [12]. It was proposed that NR4A1-dependent apoptosis is mediated through two parallel pathways: mitochondria targeting and ER targeting [11].

**Figure 1:**
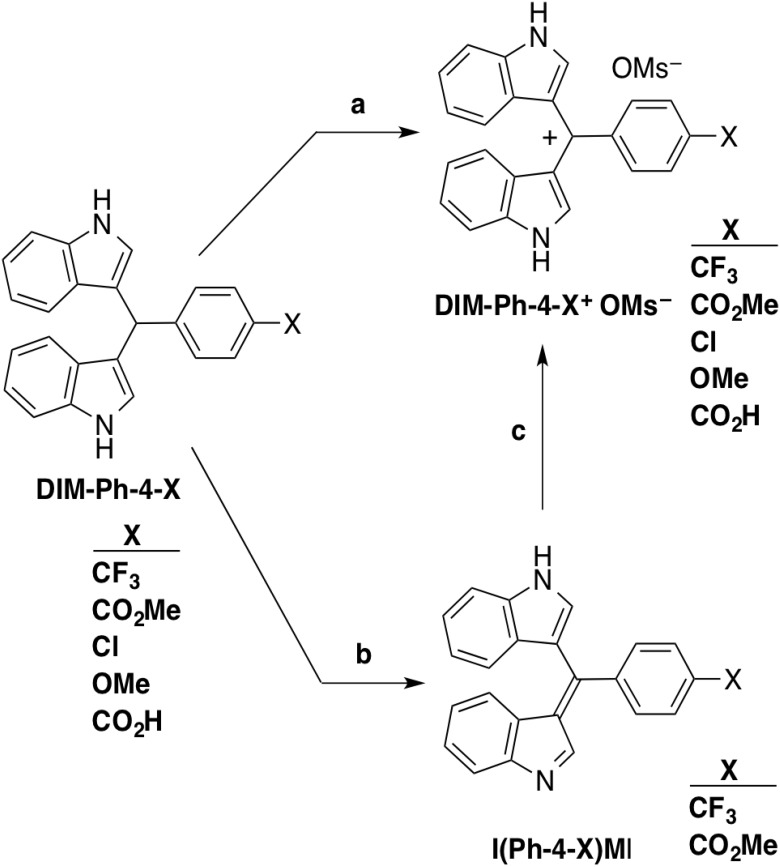
Conversion of DIM-Ph-4-Xs to their oxidation products Step a: DIM-Ph-X (X = CF_3_, CO_2_Me, Cl, OMe or CO_2_H) (0.5 mmol), 1-butanol (5 mL), powdered charcoal (10–25 mg), MsOH (1.5 mmol), stirring under air or in the presence of an O2 bubbler (X = CF3 or CO2Me) (>20 h), filtration, washing, drying and concentration afforded the DIM-Ph-4-X+ OMs– (6-27% or >30–60% yield, respectively). One tautomeric cation structure shown. Step b: DIM-Ph-X (X = CF_3_ or CO_2_Me), DDQ, MeCN. One tautomer shown. Step c: MsOH, 1-butanol (76% or 75% yield, respectively).

Despite reports that the NR4A1 ligand-binding domain (LBD) lacks a canonical NR ligand-binding pocket (LBP), crystallographic studies demonstrated ligand binding at two allosteric sites [13]. In addition, DIM-Ph-4-CF_3_ (Figure 1) transactivated NR4A1 in reporter assays, recruited co-activators, and induced expression of NR4A1 target genes [14]. DIM-Ph-4-CF_3_ inhibited cancer cell proliferation and induced apoptosis at moderate micromolar concentrations and mediated NR4A1-induced cleavage of poly(adenosyl)ribose polymerase (PARP) and other apoptosis markers [10, 14–20]. DIM-Ph-4-CF_3_ and analogs (DIM-Ph-4-Xs) inhibited the viability of bladder [19], colon [10, 15, 16], pancreatic [14], breast [20], and prostate cancer [17] cell lines and lymphoma and acute myeloid leukemia (AML) lines [18]. DIM-Ph-4-OMe reduced the tumor growth in xenograft experiments [10, 19]. NR4A1 localized in the nucleus after treatment with DIM-Ph-4-OMe and had antiproliferative activity irrespective of the presence of nuclear export inhibitor leptomycin B [14], suggesting a nuclear site of action. These results provided a basis for optimizing NR4A1 ligands as anti-cancer agents.

Resynthesizing DIM-Ph-4-CF_3_ [20] (scheme in Supplementary Figure 1A) for use as a positive control to assess analogs of NR4A1 ligand cytosporone B (Csn-B, Supplementary Figure 1B) as apoptosis inducers [21, 22] led to the discovery of an oxidation product that was more potent than DIM-Ph-4-CF_3_. Here, we describe this unreported compound, its analogs, their impact on cancer cell viability and apoptosis, and NR4A1 as a plausible target in their apoptosis induction pathway.

## RESULTS

### DIM-Ph-4-Xs undergo oxidation to DIM-Ph-4-X^+^ OMs^–^s and I(Ph-4-X)MIs (X = CF_3_ and CO_2_Me)

DIM-Ph-4-CF_3_ analogs having phenyl ring 4-substituents (X) that varied in electronic effects [23] and volumes were selected from those previously reported [24] to determine whether they would oxidize to more active species. The OMe group of DIM-Ph-4-OMe was expected to behave as an electron donor, and the CF_3_, CO_2_Me, Cl and CO_2_H groups of other DIM-Ph-4-Xs were expected to withdraw electrons [25–27]. In addition, in cell culture media, the CO_2_H group was expected to deprotonate to the carboxylate (CO_2_^–^), a moderate electron donor. Calculated group volumes ranged from 22.6–53.6 Å. DIM-Ph-4-Xs were synthesized (74–96% yields) by condensing indole with 4-X-benzaldehydes (Supplementary Figure 1A). Exposure of DIM-Ph-4-Xs to air or oxygen in the presence of MsOH and carbon powder produced the corresponding methanesulfonate (mesylate, OMs^–^) salts (DIM-Ph-4-X^+^ OMs^–^s) (Figure 1). MsOH was added to convert oxidation products to OMs^–^ salts, thereby enhancing solubility in culture medium. Oxidation of DIM-Ph-4-CF_3_ and DIM-Ph-4-CO_2_Me using DDQ produced more readily isolatable I(Ph-4-CF_3_)MI and I(Ph-4-CO_2_Me)MI in higher yields (88% and 76%, respectively) (Figure 1). Thus, DIM-Ph-4-Xs undergo oxidation under neutral conditions. MsOH treatment converted I(Ph-4-X)MIs (X = CF_3_ and CO_2_Me) to their mesylate salts (DIM-Ph-4-X^+^ OMs^–^s) (Figure 1) as shown by infrared and ^1^H and ^13^C NMR spectroscopy (see Supplementary Data) to demonstrate that protonation of I(Ph-4-X)MIs by an acid produces the corresponding salts.

**Table 1:** Effects of the DIM-Ph-4-Xs and their oxidation products, DIM-Ph-4-X^+^ OMs^–^s on the viability of human colon and breast cancer cell lines^a^ and MMTV-Wnt1 murine mammary stem cells in culture after treatment for 72 h

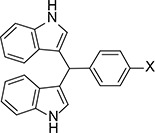	viability of treated cells relative to vehicle control alone-treated cells (IC50 value and % of control at maximum treatment concentration)b							
		HCT116 colon cancerc		MCF7 breast cancerc		MDA-MB-231 breast cancerc		MMTV-Wnt1 mammary stem cellsd	
DIM-Ph-4-X X	IC50 (μM)	% at 2.0 μM	IC50 (μM)	% at 2.0 μM	IC50 (μM)	% at 2.0 μM	IC50 (μM)	% at 3.0 μM
CF3	nd^e^	nd	>2.0	80.9 ± 5.6	>2.0	77.7 ± 3.4	1.85	1.9 ± 0.99
CO2Me	nd	nd	>2.0	96.1 ± 0.5	>2.0	98.9 ± 1.5	>3.0	99.1 ± 13.3
Cl	nd	nd	>2.0	89.6 ± 3.8	>2.0	98.1 ± 2.3	>3.0	131.5 ± 5.7
OMe	nd	nd	>2.0	95.5 ± 0.9	>2.0	99.0 ± 2.6	>3.0	122.1 ± 6.5
CO2H	nd	nd	>2.0	99.4 ± 10.2	>2.0	95.3 ± 8.7	>3.0	123.9 ± 7.5
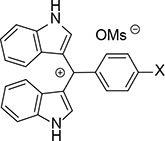 DIM-Ph-4-X+ OMs– X								
CF3	0.1	0.9 ± 0.1	0.10	–6.0 ± 0.8	0.10	4.0 ± 0.3	0.05	0.4 ± 0.1
CO2Me	0.4	4.2 ± 1.5	0.20	–5.9 ± 1.0	0.14	0.2 ± 3.4	0.14	0.3 ± 0.1
Cl	0.8	7.5 ± 1.8	0.80	17 ± 4.2	0.25	1.1 ± 0.0	0.17	0.4 ± 0.3
OMe	>2.0	73 ± 2.5	1.4	26.6 ± 6.8	1.33	0.1 ± 0.3	0.64	0.9 ± 0.2
CO2H	>2.0	102 ± 12	>2.0	98.5 ± 9.2	>2.0	104 ± 1.3	>3.0	123.2 ± 20.5

*^a^*Cancer cell line mutations are described in the supplementary data. *^b^*IC_50_ values (concentrations giving 50% viability relative to vehicle controls) were determined by interpolation of the concentration–cell viability response curves shown in Supplementary Figure 2 and using data representing means of triplicates ± SD compared to control values as were their viabilities (% of control) at the maximum concentrations tested. Viability was determined using a commercial assay for the cancer cell lines or the high-content screening of DAPI-stained stem cells as described in the Methods. *^c^*Colon and breast cancer cell lines were treated with 0.0, 0.125, 0.250, 0.500, 1.00 or 2.00 μM compound. *^d^*Murine mammary stem cells were treated with 0.0, 0.003, 0.010, 0.030, 0.100, 0.300, 1.00 or 3.00 μM compound. *^e^*nd, not determined.

### DIM-Ph-4-X^+^ OMs^–^s inhibit cancer cell viability

DIM-Ph-4-X^+^ OMs^–^s were evaluated for antiproliferative activity using HCT-116 colorectal cancer cells. Dose response curves after 72 h treatments (Supplementary Figure 2) were established to determine concentrations inhibiting viability by 50% (Table 1). DIM–Ph-4-X^+^ OMs^–^s (X = CF_3_, CO_2_Me and Cl) were most potent with respective IC_50_s of 0.1, 0.4 and 0.8 μM, whereas the IC_50_ for DIM–Ph-4-OMe^+^ OMs^–^ was >2.0 μM (73% viability at 2.0 μM) and DIM-Ph-4-CO_2_H^+^ OMs^–^ was inactive (102% viability) at 2.0 μM. DIM-Ph-4-CF_3_^+^ OMs^–^ at 2.0 μM inhibited growth by 99% and DIM-Ph-4-CO_2_Me^+^ OMs^–^ and DIM-Ph-4-Cl^+^ OMs^–^ produced 96% inhibition. DIM-Ph-4-CO_2_H^+^ OMs^–^ was inactive.

These results suggested evaluating DIM-Ph-4-X^+^ OMs^–^s and DIM-Ph-4-Xs using various human cancer cell lines. IC_50_s after 72 h treatments of MCF-7 and MDA-MB-231 breast cancer cells (Supplementary Figure 2) are listed in Table 1. Four oxidation products were more active than their precursors having the inhibitory potency order: X = CF_3_ ≥ CO_2_Me > Cl > OMe. The first three DIM-Ph-4-X^+^ OMs^–^s had submicromolar IC_50_ values, whereas those for DIM-Ph-4-OMe^+^ OMs^–^ were 1.0–2.0 μM. DIM-Ph-4-CO_2_H^+^ OMs^–^ was inactive at 2.0 μM. Except for DIM-Ph-4-CF_3_, which at 2.0 μM inhibited viability by 20–26%, other DIM-Ph-4-Xs were inactive.

Evaluations were extended to prostate cancer cell lines. Dose response curves showed effects on four prostate cancer lines and IC_50_ values (Table 2, Supplementary Figure 2) indicated that DIM-Ph-4-X^+^ OMs^–^s (X = CF_3_, CO_2_Me and Cl) were the most robust inhibitors (IC_50_s 0.07–0.66 μM) and were followed by DIM-Ph-4-OMe^+^ OMs^–^ (IC_50_s 0.85–1.65 μM). At 2.0 μM, inhibition by DIM-Ph-4-CO_2_H^+^ OMs^–^ was 0–1% and that by DIM-Ph-4-CF_3_ was weak (17–36%). Other DIM-Ph-4-Xs were inactive.

**Table 2:** Effects of DIM-Ph-4-Xs and their oxidation products, DIM-Ph-4-X^+^ OMs^–^s, on reducing prostate cancer cell line^a^ viability after treatment for 72 h

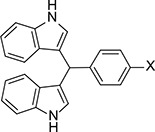	viability of treated cells relative to vehicle-alone treated cells (IC_50_ value and % of control at maximum treatment concentration)*^b^*							
	LNCaP		LAPC4		22Rv1		PC3	
DIM-Ph-4-X X	IC_50_ (μM)	% at 2.0 μM	IC_50_ (μM)	% at 2.0 μM	IC_50_ (μM)	% at 2.0 μM	IC_50_ (μM)	% at 2.0 μM
CF_3_	>2.0	82.7 ± 23.7	>2.0	77.8 ± 3.0	>2.0	63.6 ± 9.5	>2.0	72.4 ± 10.7
CO_2_Me	>2.0	89.0 ± 12.7	>2.0	106.7 ± 2.0	>2.0	105.2 ± 2.8	>2.0	101.0 ± 4.3
Cl	>2.0	91.5 ± 14.3	>2.0	101.5 ± 2.0	>2.0	104.1 ± 7.2	>2.0	101.4 ± 8.1
OMe	>2.0	91.2 ± 7.4	>2.0	104.4 ± 4.2	>2.0	108.7 ± 2.4	>2.0	104.9 ± 1.8
CO_2_H	>2.0	99.2 ± 9.3	>2.0	100.7 ± 4.1	>2.0	93.6 ± 7.2	>2.0	103.7 ± 6.4
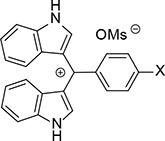 DIM-Ph-4-X^+^ OMs^–^ X								
CF_3_	0.18	1.2 ± 0.6	0.13	0.9 ± 0.0	0.07	2.3 ± 0.1	0.16	0.1 ± 0.1
CO_2_Me	0.17	0.6 ± 0.6	0.15	1.1 ± 0.2	0.17	1.7 ± 0.5	0.33	3.7 ± 0.5
Cl	0.37	0.5 ± 1.2	0.58	3.8 ± 2.6	0.38	1.0 ± 0.9	0.66	4.7 ± 1.6
OMe	0.85	20.1 ± 13.5	1.29	32.3 ± 2.9	1.45	28.0 ± 2.1	1.65	37.7 ± 3.2
CO_2_H	>2.0	93.8 ± 6.5	>2.0	98.6 ± 2.2	>2.0	89.6 ± 9.5	>2.0	95.8 ± 2.6

*^a^*Prostate cancer cell line mutations are described in the supplementary data. *^b^*Cell lines were treated with 0, 0.125, 0.25, 0.5, 1.0, or 2.0 μM compound. IC_50_ values (concentration affording 50% viability compared to vehicle control) were determined from the concentration–viability response curves shown in Supplementary Figure 2 using data representing means of triplicates ± SD, as was viability (%) at 2.0 μM.

DIM-Ph-4-CF_3_^+^ OMs^–^ and DIM-Ph-4-CF_3_ were also evaluated against seven leukemia cell lines—KG-1, MOLM-13, OCI-AML-2 and 3 and THP-1 AML, K562 CML and MOLT-4 T-cell acute lymphoid leukemia (T-ALL). Concentration–response curves after 24-h treatments (Supplementary Figures 3 and 4) were used to derive IC_50_ values and cell viabilities at 2.0 μM (AML) or 1.0 μM (CML and T-ALL) (Table 3). Viability of six lines was efficiently inhibited by DIM-Ph-4-CF_3_^+^ OMs^–^ (IC_50_s 0.73–1.0 μM), whereas the THP-1 line was more resistant (IC_50_ 1.4 μM). At 2.0 μM DIM-Ph-4-CF_3_^+^ OMs^–^ reduced AML viability by 92–100%, whereas DIM-Ph-4-CF_3_ reduced viability by 3–23% (Supplementary Figure 3).

**Table 3:** Effects of DIM-Ph-4-CF_3_ and DIM-Ph-4-CF_3_^+^ OMs^–^ on viability of seven leukemia cell lines^a^: Concentrations required to reduce viability by 50% and viability inhibition at 2.0 μM (KG-1, MOLM-13, OCI-AML-2, OCI-AML-3 and THP-1) or 1.0 μM (K562 and MOLT-4) compared to vehicle controls after 24-h treatments

compound	IC_50_ (μM)*^b^* (% viability relative to control at highest concentration tested)						
	KG-1 AML*^c^*	MOLM-13 AML*^c^*	OCI-AML-2 AML*^c^*	OCI- AML-3AML*^c^*	THP-1 AML*^c^*	K562 CML*^d^*	MOLT-4 T-ALL*^d^*
DIM-Ph-4-CF_3_	2.0 (97 ± 11)	>2.0 (77 ± 5)	>2.0 (95 ± 2)	>2.0 (82 ± 9)	>2.0 (97.9 ± 4.7)	>1.0 (87 ± 10)	>1.0 (72 ± 8)
DIM-Ph-4-CF_3_^+^ OMs^–^	0.89 (>0.1 ± 0.5)	0.73 (8.0 ± 0.2)	0.96 (0.6 ± 0.1)	0.72 (1.2 ± 0.4)	1.41 (–0.0 ± 0.0)	0.76 (13 ± 1)	0.79 (9.3 ± 0.2)

*^a^*See Supplementary Table 3 for a characteristics of the leukemia lines. *^b^*IC_50_ values were determined from the concentration–viability response curves shown in Supplementary Figure 4 and derived from data representing means of triplicates ± SD as determined by the viability assay described in the Methods. *^c^*AML cell lines were treated at 0.0, 0.10, 0.25, 0.50, 1.0 or 2.0 μM compound. *^d^*CML and T-ALL leukemia lines were treated at 0.0, 0.0625, 0.125, 0.25, 0.50 or 1.00 μM compound.

Cancer cell proliferative activity has been attributed to stem cell-like characteristics. NR4A1 expression is up-regulated in gastric cancer cells with higher NR4A1 levels correlating with stem-like properties of cells grown as tumor spheres [28]. Therefore, DIM-Ph-4-X^+^ OMs^–^s and DIM-Ph-4-Xs were evaluated for inhibiting proliferation of MMTV-Wnt1 murine mammary cancer stem cells [29]. Again, DIM-Ph-4-CF_3_^+^ OMs^–^ was most potent (IC_50_ 50 nM) having 37-fold higher activity than DIM-Ph-4-CF_3_ (Supplementary Figure 5 and Table 1). DIM-Ph-4-CO_2_Me^+^ OMs^–^ and DIM-Ph-4-Cl^+^ OMs^–^ were less active (IC_50_s 140 nM and 170 nM, respectively) and were followed by DIM-Ph-4-OMe^+^ OMs^–^ (IC_50_ 640 nM). DIM-Ph-4-CO_2_H^+^ OMs^–^ and DIM-Ph-4-Xs (X = CO_2_Me, Cl, OMe and CO_2_H) were inactive. This data shows that the oxidation products DIM-Ph-4-X^+^ OMs^–^s also inhibit the growth of cancer stem cells.

### DIM-Ph-4-CF_3_^+^ OMs^–^ induce apoptosis

**Figure 2:**
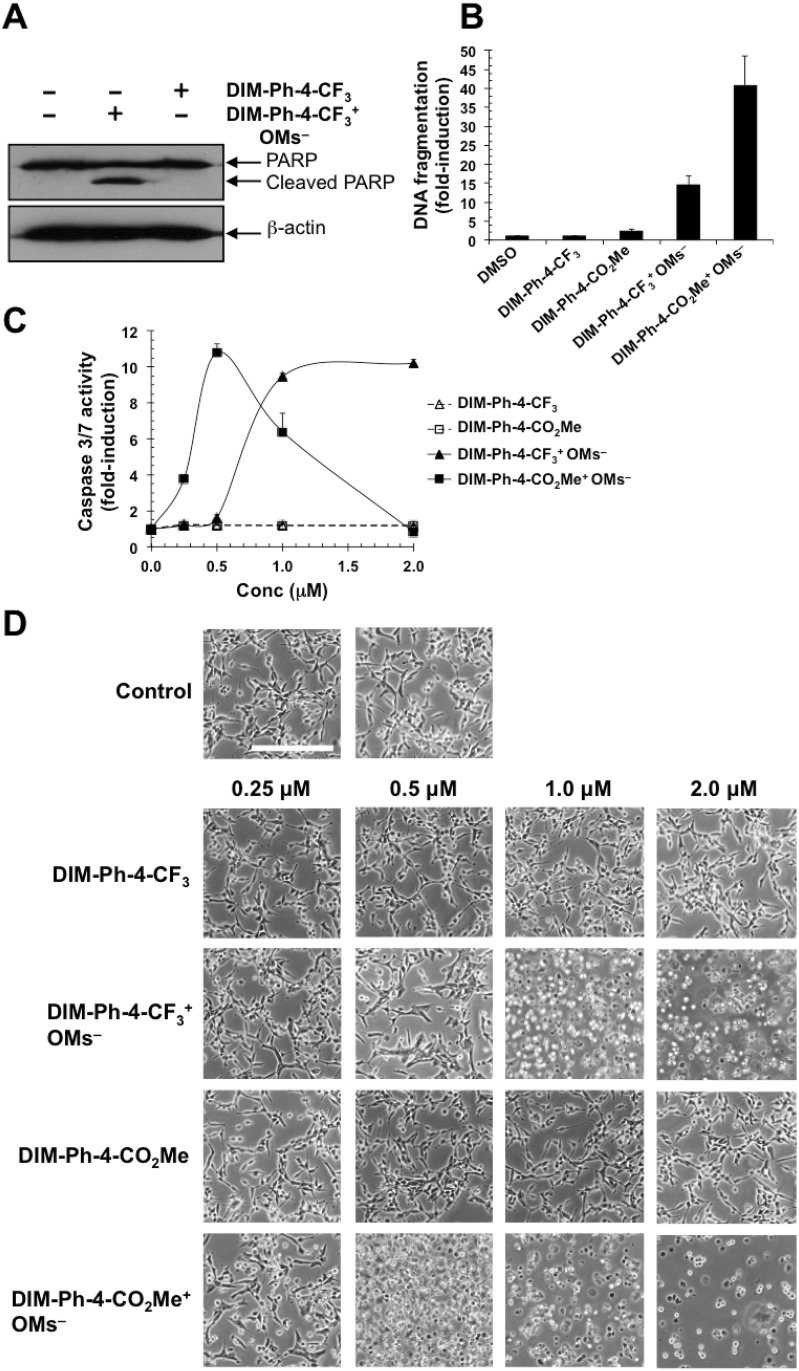
Effect of DIM-Ph-4-CF_3_^+^ OMs^–^ and DIM-Ph-4-CO_2_Me^+^ OMs^–^ on cancer cell viability (**A**) Treatment of HCT-116 colon cancer cells with DIM-Ph-4-CF_3_^+^ OMs^–^ induces PARP cleavage, whereas DIM-Ph-4-CF_3_ does not. Cells were treated at 1.0 μM for 6 h in medium containing 10% FBS. Apoptosis was determined by Western blotting for PARP cleavage using β-actin as a reference. (**B**) LNCaP prostate cancer cells were exposed to 2.0 μM of the indicated compounds for 24 hours and DNA fragmentation was determined as described in Methods (average of two replicates). (**C**) LNCaP cells were treated with compounds for 24 hours at increasing concentrations (0.25, 0.5, 1.0, 2.0 μM), and caspase 3/7 activation was measured (means ± SD of triplicates or quadruplicates). The decline in caspase 3/7 activity at doses of DIM-Ph-4-CO_2_Me^+^ OMs^–^ above 5 μM is due to cell loss as a result of excessive cell death. (**D**) LNCaP cells were treated with the indicated compounds for 24 hours, followed by fixation and microscopic examination. Calibration bar in upper left panel represents 100 μm.

Because DIM-Ph-4-CF_3_ was reported to induce cancer cell apoptosis and PARP cleavage [14], we investigated whether DIM-Ph-4-CX^+^ OMs^–^s induced apoptosis. After 6 h, PARP cleavage in HCT-116 cancer cells treated with 1.0 μM DIM-Ph-4-CF_3_^+^ OMs^–^ and DIM-Ph-4-CF_3_ were compared. DIM-Ph-4-CF_3_^+^OMs^–^ induced cleavage, whereas DIM-Ph-4-CF_3_ did not (Figure 2A).

DIM-Ph-4-CF_3_^+^ OMs^–^ and DIM-Ph-4-CO_2_Me^+^ OMs^–^ were also evaluated using other apoptosis markers. LNCaP cells were treated at 2.0 μM for 24 h before DNA fragmentation was measured (Figure 2B). DIM-Ph-4-CF_3_^+^ OMs^–^ and DIM-Ph-4-CO_2_Me^+^ OMs^–^ increased fragmentation by 14- and 40-fold, respectively, whereas DIM-Ph-4-CF_3_ with a 0.96-fold fragmentation change was considered inactive, and DIM-Ph-4-CO_2_Me with a 2.0-fold increase was considered to have low activity.

Caspase-3 activation was evaluated next. DIM-Ph-4-Xs lacked activity, whereas DIM-Ph-4-X^+^ OMs^–^s were active (Figure 2C). Interestingly, these mesylates produced different response curves. Cell death was also examined qualitatively by microscopic visualization of LNCaP cells after 24 h (Figure 2D). DIM-Ph-4-CF_3_^+^ OMs^–^ and DIM-Ph-4-CO_2_Me^+^ OMs^–^ at ≥1.0 μM and ≥0.5 μM, respectively, induced apoptosis, whereas DIM-Ph-4-CF_3_ and DIM-Ph-4-CO_2_Me were inactive at 2.0 μM.

### DIM-Ph-4-CF_3_^+^ OMs^–^ interacts with the NR4A1 LBD

**Figure 3:**
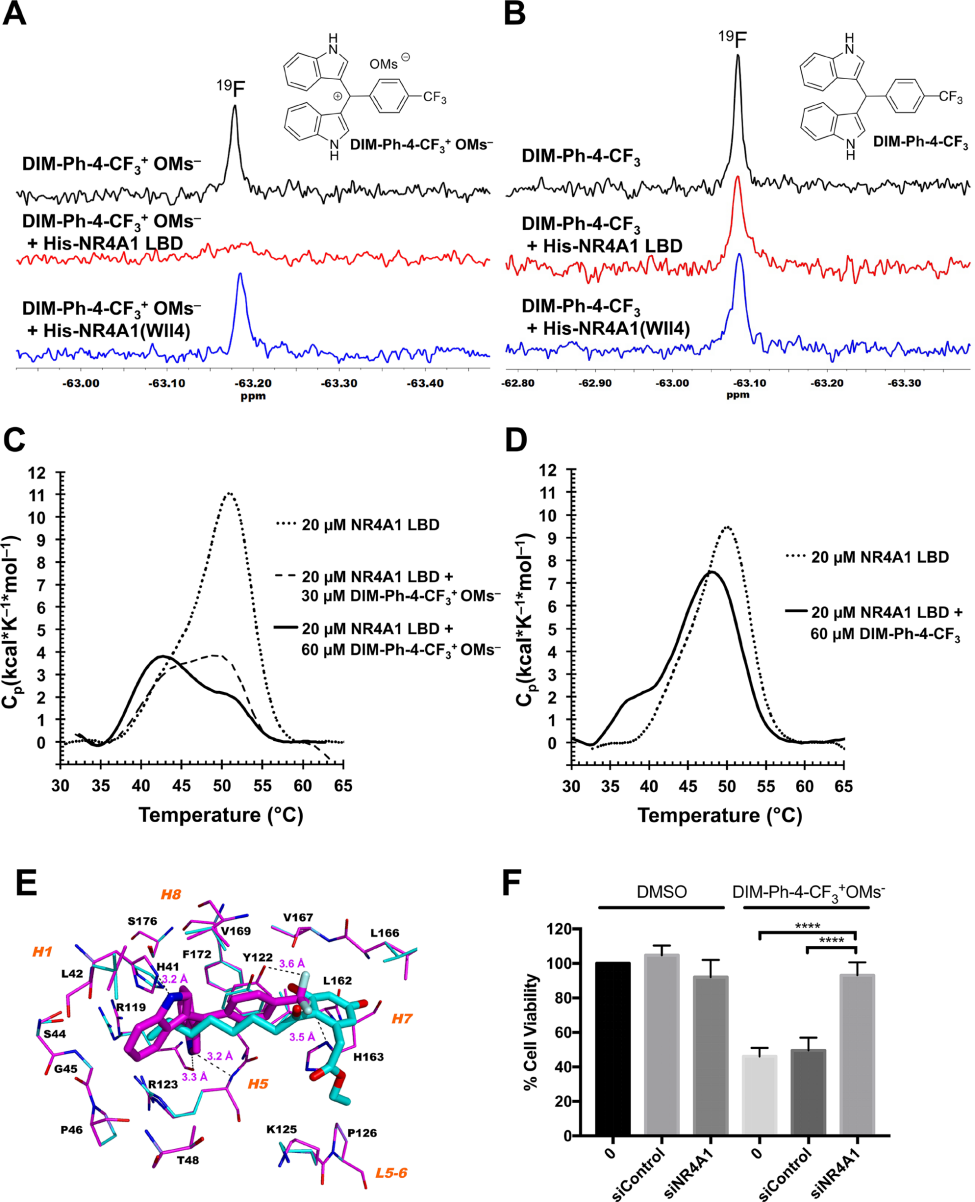
DIM-Ph-4-CF_3_^+^ OMs^–^ binds to the NR4A1 LBD (**A**) High-field region of ^19^F NMR spectra of 500 μM DIM-Ph-4-CF_3_^+^ OMs alone (upper spectrum in black) and with 5.0 μM recombinant NR4A1 (TR3) LBD protein (middle spectrum in red) or with NR4A1 N-terminal region mutant lacking the LBD domain (TR3(WII4)) (lower spectrum in blue) at 11° C. Spectra were recorded using 128 transients, a 14,124-Hz sweep width and a 3-sec repetition time on a 500-MHz Advance NMR spectrometer (Bruker) having a fluorine probe. (**B**) ^19^F NMR signal for DIM-Ph-4-CF_3_ (upper spectrum in black) was not lost in the presence of NR4A1 LBD (middle spectrum in red) under the same conditions as in (A) and had comparable amplitude to that observed in presence of mutant protein (lower spectrum in blue). (**C** and **D**) Differential scanning calorimetry demonstrates DIM-Ph-4-CF_3_^+^ OMs^–^–NR4A1 LBD interaction. Samples contained NR4A1 LBD (20 μM, 0.51 mg/ml) alone or with compound (30 or 60 μM) in 5% DMSO in PBS, pH 7.4, or 5% DMSO in PBS alone (reference sample). Scans were conducted at 1 K/min under 3.0-atm pressure. (**C**) 20 μM LBD alone: T_m_ = 51.5° C, ΔH = 101 kcal/mol (dashed line); 20 μM LBD with 30 μM DIM-Ph-4-CF_3_^+^ OMs^–^: T_m_ = 49.3° C, ΔH = 42 kcal/mol (dotted line); 20 μM LBD with 60 μM DIM-Ph-4-CF_3_^+^ OMs^–^: T_m_ = 43.4° C, ΔH = 44 kcal/mol (solid line). Results indicate LBD protein is destabilized by DIM-Ph-4-CF_3_^+^ OMs^–^. (**D**) 20 μM LBD alone: T_m_ = 50.1° C, ΔH = 83 kcal/mol (dashed line); 20 μM LBD with 60 μM DIM-Ph-4-CF_3_: T_m_ = 48.6° C, ΔH = 81 kcal/mol (solid line). Supplementary data provides detailed methods. T_m_, maximum thermal transition temperature; ΔH, calorimetric enthalpy calculated as area under excess heat capacity function; C_p_, excess heat capacity function. (**E**) DIM-Ph-4-CF_3_^+^ of DIM-Ph-4-CF_3_^+^ OMs^–^ docks to the NR4A1 LBD protein structure. Low-energy conformations of DIM-Ph-4-CF_3_^+^ (Cs in magenta) and NR4A1 ligand Csn-B (Cs in cyan) dock to the allosteric site on the apo-NR4A1 LBD crystal structure (PDB 2QW4). The surrounding binding site backbones and side chain Cs are depicted in magenta and cyan, respectively. Docked poses were aligned by superposing LBD backbones. Binding site residues within 6 Å of ligand are labeled using one letter format for clarity, as are helices (*H*) and loops (*L*). Fs are light-blue; Ns, blue; and Os, red. Potential H-bond interactions are denoted by dashed black lines with interatom distances given in Å and include DIM-Ph-4-CF_3_^+^ NHs with binding-site atoms *H*1 H41 NH, *H*5 R119 C=O and *H*5 R123 backbone NH; and DIM-Ph-4-CF_3_^+^ Fs with *H*5 Y122 OH and *H*7 H163 NH. Human NR4A1 LBD residue numbering is used. H atoms are omitted for clarity. Docking employed BioMed Cache software version 6.2. (**F**) LNCaP cells were transfected with either control siRNA or siRNA targeting NR4A1 for 48 hrs. Cells were then treated with either DMSO or 10 μM DIM-Ph-4-CF_3_^+^ OMs^–^ for 24 hrs and an MTT assay was performed. Statistical analysis was assessed with an unpaired *t* test. *P* values ≤ 0.0001.

Csn-B and ethyl 2-[2,3,4-trimethoxy-6-(1-octanoyl)phenyl]acetate (Supplementary Figure 1B) are known to interact with the NR4A1 LBD, and Csn-B activates NR4A1 and induces apoptosis in gastric cancer cells (64% at 15 μM after 48 h) [22]. Thus, we used nuclear magnetic resonance (NMR) spectroscopy to determine whether DIM-Ph-4-CF_3_^+^ OMs^–^ interacted with the NR4A1 LBD. This method was selected to accommodate the mesylate at its maximum aqueous solubility (~50 μM) and limited functional protein. We had validated this method by comparing NMR proton spectra of Csn-B alone and in the presence of NR4A1 LBD protein [21]. Because overlapping proton signals from protein and media complicate spectral interpretations, use of ^19^F signals (–63.2 ppm) due to a ligand circumvents this problem. ^19^F spectra of DIM-Ph-CF_3_^+^ OMs^–^ alone and with LBD protein (Figure 3A), black and red spectra, respectively) indicate that the ^19^F atom singlet for DIM-Ph-CF_3_^+^ OMs^–^ was suppressed by the LBD to indicate interaction, whereas the mutant NR4A1(WII4) lacking the LBD was unable to suppress this signal (Figure 3A, blue spectrum). These results indicate that DIM-Ph-CF_3_^+^ OMs^–^ interacts specifically with the LBD. In contrast, the ^19^F singlet in the spectrum of DIM-Ph-4-CF_3_ taken in the presence of the NR4A1 LBD was not lost and resembled that obtained in the presence of mutant protein (Figure 3B). Thus, NMR suggests that DIM-Ph-4-CF_3_^+^ OMs^–^ rather than DIM-Ph-4-CF_3_ is the major species interacting with the LBD. Differential scanning calorimetry (DSC) results (Figure 3C and 3D) support the NMR results. Addition of DIM-Ph-CF_3_^+^ OMs^–^ destabilized the NR4A1 LBD as indicated by reduced T_m_ and C_p_ values compared to those of the apo-LBD. In contrast, DIM-Ph-CF_3_ produced smaller reductions, indicating lower binding.

Using wild-type and mutant NR4A1 LBDs, Csn-B was found to bind to a surface-accessible allosteric pocket (Site A) in the LBD (PDB 2QW4) [22]. The pocket was confirmed using the crystal structure of the NR4A1 LBD bound with the Csn-B analog and NR4A1 antagonist ethyl 2-[2,3,4-trimethoxy-6-(1-octanoyl)phenyl]acetate (PDB 3V3Q) [13]. Structure 3V3Q reveals that the LBD bound a second molecule in an even more surface-accessible pocket (Site B). We first conducted docking of the cation (DIM-Ph-4-CF_3_^+^) of DIM-Ph-4-CF_3_^+^ OMs^–^ to Site A on the apo-NR4A1 LBD (2QW4), which we had verified for Cns-B [21]. Overlap of our docked poses for DIM-Ph-4-CF_3_^+^ and Cns-B suggested that one DIM-Ph-4-CF_3_^+^ F could interact with helix *H*5 Tyr122 OH and another F with *H*7 His163 ring NH (Figure 3E). A F in a C*sp_3_*–F bond has been reported to function as a weak H-bond acceptor [30]. DIM-Ph-4-CF_3_^+^ indole ring NHs could H-bond with *H*1 His41 ring NH and *H*5 Arg119 C=O and Arg123 backbone NH. Next, we docked DIM-Ph-4-CF_3_^+^ to both allosteric sites on the NR4A1 LBD–Csn B analog (3V3Q). Its docked pose in Site A was similar to that obtained by docking to the apo-LBD (2QW4), although one indole ring was not buried as deeply in the cleft and the other indole interacted with Pro46 rather than His41 (Supplementary Figure 6A). Docking of DIM-Ph-4-CF_3_^+^ into Site B provided a pose in which both indole rings were exposed on the LBD surface and the CF_3_ extended into the cleft towards *H*3 while its phenyl ring lay adjacent to a surface ridge formed by *H*4, *H*11 and *H*12 residues (Supplementary Figure 6B).

Given our observation that DIM-Ph-4-CF_3_^+^ OMs^–^ interacted with NR4A1, we asked whether NR4A1 is required for DIM-Ph-4-CF_3_^+^OMs^–^-induced apoptosis. To this end, NR4A1 was knocked down in LNCaP-SKP2 cells (cell line described below) (Supplementary Figure 8D), and cells treated with DIM-Ph-4-CF_3_^+^OMs^–^ for 24 hours. An MTT assay was performed to measure cell viability (Figure 3F). While cell viability was decreased in untransfected and control siRNA transfected cells, DIM-Ph-4-CF_3_^+^OMs^–^ did not have a cytotoxic effect on cells when NR4A1 was downregulated. In summary, these results indicate that DIM-Ph-4-CF_3_^+^OMs^–^ induces apoptosis of cancer cells through specific binding to NR4A1.

### DIM-Ph-4-CF_3_^+^OMs^-^ inhibits prostate cancer cell growth *in vivo*

**Figure 4:**
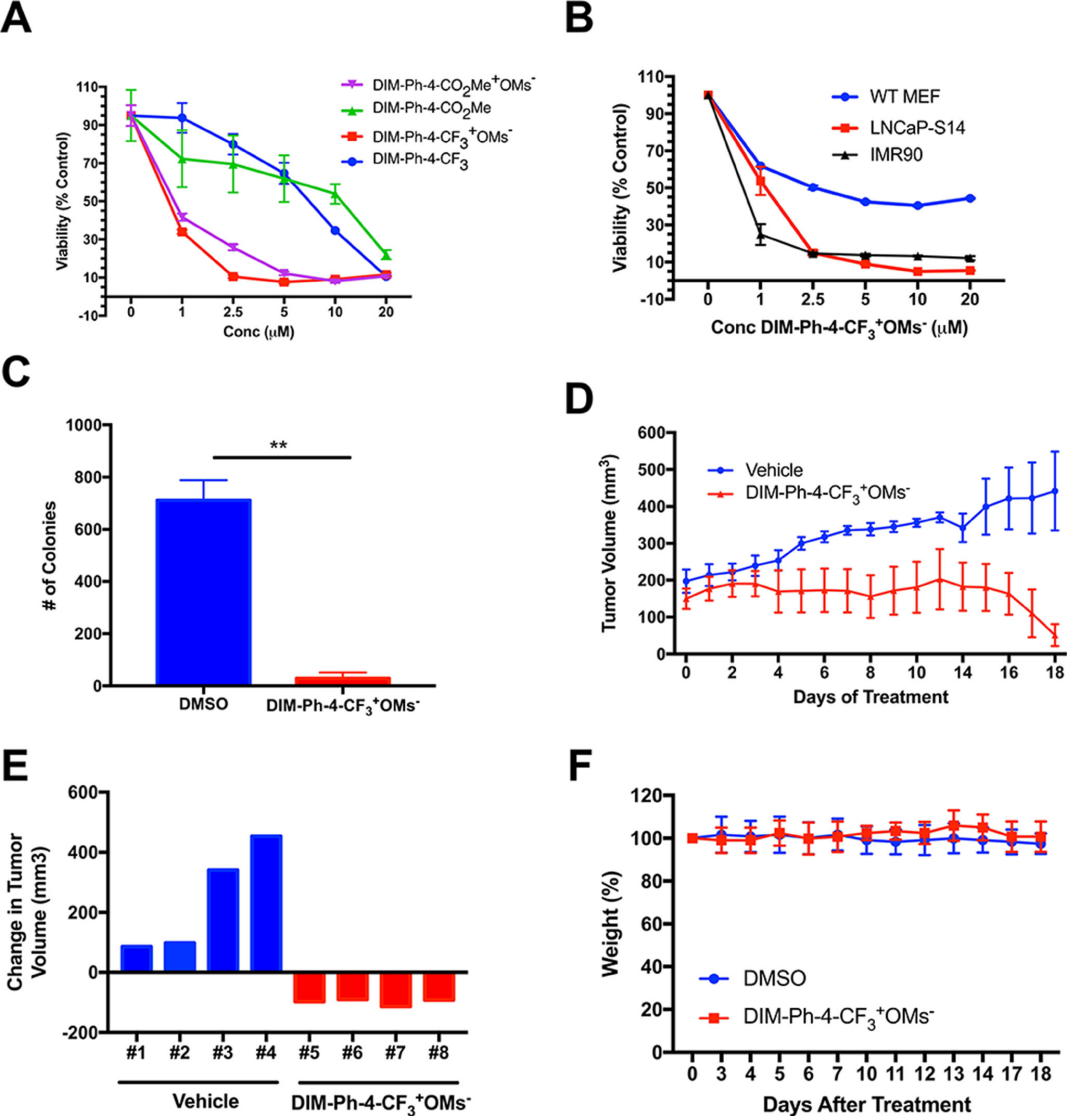
DIM-Ph-4-CF_3_^+^OMs^-^ inhibits prostate cancer growth *in vivo* (**A**) LNCaP-SKP2 cells were treated with DIM-Ph-4-CO_2_Me^+^OMs^-^, DIM-Ph-4-CO_2_Me, DIM-Ph-4-CF_3_^+^OMs^-^ or DIM-Ph-4-CF_3_ at specified concentrations for 72 hours (*n* = 8). Cell viability was measured by MTT assay to determine the cytotoxic potential of each compound. (**B**) LNCaP-SKP2 cells, WT mouse embryonic fibroblasts and IMR90 cells were treated with either DMSO or DIM-Ph-4-CF_3_^+^OMs^-^ at specified concentrations for 72 hours (*n* = 8). Cell viability was measured by MTT assay to assess selectivity. (**C**) The graph represents clonogenic assays (*n* = 2) performed with LNCaP-SKP2 cells and treated once a week for 3 weeks with either DMSO or DIM-Ph-4-CF_3_^+^OMs^-^ (2 uM). (**D**) LNCaP-SKP2 xenografts were grown in NOD/SCID mice. Four animals received DIM-Ph-4-CF_3_^+^OMs^-^ (15 mg/kg i.p.) for 18 days while the remaining four mice were treated with vehicle. The graph represents mean tumor volumes ± standard deviations in each group over time. (**E**) The response of DIM-Ph-4-CF_3_^+^OMs^-^ (15 mg/kg) or vehicle *in vivo* for individual NOD/SCID mice was expressed as change in tumor volume (day 18 minus day 0). (**F**) The graph represents relative average body weights of NOD/SCID mice ± standard deviations in the DIM-Ph-4-CF_3_^+^OMs^-^ treated and DMSO control groups over 18 days of treatment.

In order to assess the therapeutic potential of select compounds, we tested the *in vitro* and *in vivo* efficacy of DIM-Ph-4-CF_3_^+^OMs^-^ on human prostate cancer cells. LNCaP-SKP2 cells were used for both *in vitro* and *in vivo* studies. The LNCaP-SKP2 line was derived by stably overexpressing the SKP2 subunit of the CRL1^SKP2^ ubiquitin ligase in human LNCaP prostate cancer cells. As a result of SKP2 overexpression, LNCaP-SKP2 cells exhibited downregulation of the cyclin-dependent kinase inhibitor p27, a hallmark of aggressive prostate cancer (Supplementary Figure 7). The oxidation products DIM-Ph-4-CF_3_^+^OMs^-^ and DIM-Ph-4-CO_2_Me^+^OMs^-^ had a greater effect on LNCaP-SKP2 viability than DIM-Ph-4-CO_2_Me and DIM-Ph-4-CF_3_, causing a 90% reduction in relative cell viability (Figure 4A). Since DIM-Ph-4-CF_3_^+^OMs^-^ demonstrated a higher potency, it was further evaluated *in vitro* for selectivity. Treatment of wildtype mouse embryonic fibroblasts, human IMR90 fibroblasts and LNCaP-SKP2 cells with DIM-Ph-4-CF_3_^+^OMs^-^ resulted in a greater decrease in cell viability in LNCaP-SKP2 cells than the MEFs even though IMR90 cell viability was substantially decreased (Figure 4B). In addition, DIM-Ph-4-CF_3_^+^OMs^-^ significantly inhibited LNCaP-SKP2 cell colony forming ability as demonstrated by clonogenicity assay (Figure 4C).

In order to confirm the inhibitory effect of DIM-Ph-4-CF_3_^+^OMs^-^, *in vivo* studies were conducted in a murine xenograft model. We first determined the maximally tolerated dose of DIM-Ph-4-CF_3_^+^OMs^-^ (25 mg/kg intraperitonially, i.p.; data not shown). NOD/SCID mice bearing LNCaP-SKP2 tumors were dosed with 15 mg/kg i.p. daily. DIM-Ph-4-CF_3_^+^OMs^-^ potently suppressed tumor growth as judged by average tumor volumes (Figure 4D). DIM-Ph-4-CF_3_^+^OMs^-^ led to tumor shrinkage in all four animals, while vehicle control treated mice showed an increase in tumor volume over time (Figure 4E). Only insignificant weight loss was observed (Figure 4F). Collectively, both *in vivo* and *in vitro* results demonstrate that DIM-Ph-4-CF_3_^+^OMs^-^ selectively inhibits prostate cancer cells without apparent toxicity in a rodent model.

### DIM-Ph-4-CF_3_^+^ OMs^–^ and DIM-Ph-4-CO_2_Me^+^ OMs^–^ induce the unfolded protein response

**Figure 5:**
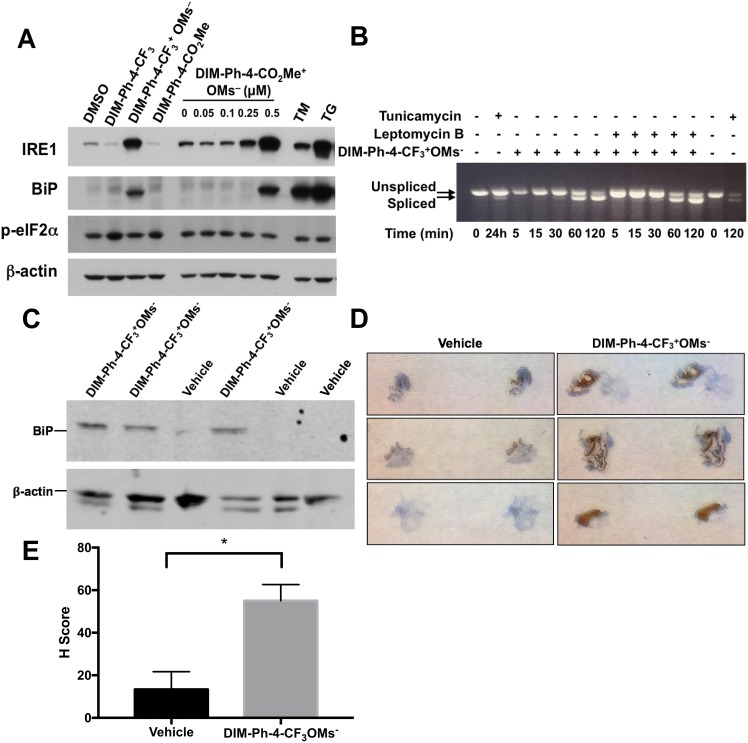
Effect of DIM-Ph-4-CF_3_^+^ OMs^–^ and DIM-Ph-4-CO_2_Me^+^ OMs^–^ on the unfolded protein response (**A**) DIM-Ph-4-CF_3_^+^ OMs^–^ and DIM-Ph-4-CO_2_Me^+^ OMs^–^ induce the unfolded protein response (UPR) in LNCaP prostate cancer cells, whereas DIM-Ph-4-CF_3_ and DIM-Ph-4-CO_2_Me do not. Total extracts from cells treated for 24 hours with 2.0 μM DIM-Ph-4-CF_3_, 2.0 μM DIM-Ph-4-CF_3_^+^ OMs^–^, or 2.0 μM DIM-Ph-4-CO_2_Me in DMSO respectively. Separately, cells were treated for 24 hours with 0.05 to 0.50 μM DIM-Ph-4-CO_2_Me^+^ OMs^–^ in DMSO, or DMSO alone (0.1% final concentration). Immunoblotting was used to analyze expression of UPR markers—IRE1a (IRE1), BiP/GRP78 (BiP) and phospho-eIF2α (p-eIF2α)—using β-actin as the loading control as described in Methods. Tunicamycin (TM, 1.0 μM) and thapsigargin (TG, 1.0 μM) were used as UPR-inducing controls. Figure is representative of two independent experiments. (**B**) Kinetics of XBP1 splicing as another marker of UPR induction was analyzed by RT-PCR with total RNA from LNCaP-SKP2 cells treated with 10 μM DIM-Ph-4-CF_3_^+^OMs^-^ for increasing time points from 5 to 120 minutes. Where indicated, cells were pre-treated with Leptomycin B (50 nM) for 1 hour prior to DIM-Ph-4-CF_3_^+^OMs^-^ treatment. Tunicamycin (TM, 5 μg/mL) was used as a UPR-inducing control. (**C**) UPR induction was analyzed *in vivo* in LNCaP-SKP2 xenografts from mice treated with either vehicle control or 10 mg/kg DIM-Ph-4-CF_3_^+^OMs^-^. Once tumors were 200-200 mm^3^, mice were treated every 12 hrs for 24 hrs. Tumors were harvested and protein was analyzed by Western blot for BiP expression. (**D**) LNCaP-SKP2 xenografts from mice treated with either vehicle or 10 mg/kg DIM-Ph-4-CF_3_^+^OMs^-^ every 12 hrs for 24 hrs were harvested and BiP expression was analyzed by immunohistochemistry. (**E**) H scores for immunohistochemistry staining were calculated as described in the methods. Statistical significance was analyzed with an unpaired *t* test (*p* value = 0.0211).

NR4A1 has been implicated in endoplasmic reticulum (ER) stress-induced apoptosis [11]. DIM-Ph-4-Br and DIM-Ph-4-F at 15 μM induced ER stress-associated apoptosis [31]. Therefore, we examined whether DIM-Ph-4-CF_3_, DIM-Ph-CO_2_Me, DIM-Ph-4-CF_3_^+^ OMs^–^ and DIM-Ph-4-CO_2_Me^+^ OMs^–^ induced the ER-associated unfolded protein response (UPR) in LNCaP cells using the ER stress markers IRE1, BiP/GRP78 and phosphorylated eIF2α (p-eIF2α). Similar to 1.0 μM of the classical UPR inducers thapsigargin (TG) and tunicamycin (TM), 2.0 μM DIM-Ph-4-CF_3_^+^ OMs^–^ and 0.5 μM DIM-Ph-4-CO_2_Me^+^ OMs^–^ induced robust IRE1 and BiP/GRP78 expression at 24 h, whereas levels induced by 2.0 μM DIM-Ph-4-CF_3_ and DIM-Ph-CO_2_Me were very low (Figure 5A). Induction of p-eIF2α by either mesylate, TG or TM was not detected under our conditions. Additionally, splicing of transcription factor XBP1 mRNA was evaluated as another UPR indicator. DIM-Ph-4-CF_3_^+^OMs^-^ induced XBP1 splicing as early as 30 minutes after treatment, and the ratio of spliced to unspliced mRNA continued to increase within 2 hours of treatment (Figure 5B). UPR induction was also observed *in vivo* through the upregulation of BiP expression in LNCaP-SKP2 xenografts grown in mice treated with DIM-Ph-4-CF_3_^+^OMs^-^ (Figure 5C–5E).

### The subcellular localization of NR4A1 is consistent with an extranuclear mechanism-of-action of DIM-Ph-4-CF_3_^+^OMs^-^

We noticed that DIM-Ph-4-CF_3_^+^OMs^-^-mediated induction of XBP1 splicing was retained in cells pre-treated with the nuclear export inhibitor Leptomycin B, indicating that it did not require redistribution of NR4A1 from the nucleus to the cytoplasm (Figure 5B). To begin to address whether DIM-Ph-4-CF_3_^+^OMs^-^ stimulated NR4A1 exerts its pro-apoptotic function in the nucleus or in the cytoplasm, we determined the sub-cellular partitioning of NR4A1 by immunofluorescence staining. Using siRNA-mediated knockdown, we first confirmed that the commercial rabbit NR4A1 XP**^®^** MAb (Cell Signaling Technology) antibody recognized endogenous NR4A1 in LNCaP-SKP2, MCF7 and 293T cells (Supplementary Figure 8). By immunofluorescence staining, we found that a substantial portion of NR4A1 was localized to the extranuclear compartment of all three cell lines (Supplementary Figure 8A–8C). This localization pattern was confirmed by immunofluorescence staining with another commercial rabbit NR4A1 polyclonal antibody (Sigma-Aldrich) antibody (Supplementary Figure 8A–8C) and by immunoblotting of nuclear and cytoplasmic fractions isolated from LNCaP-SKP2 and MCF7 cells (Supplementary Figure 8E).

**Figure 6:**
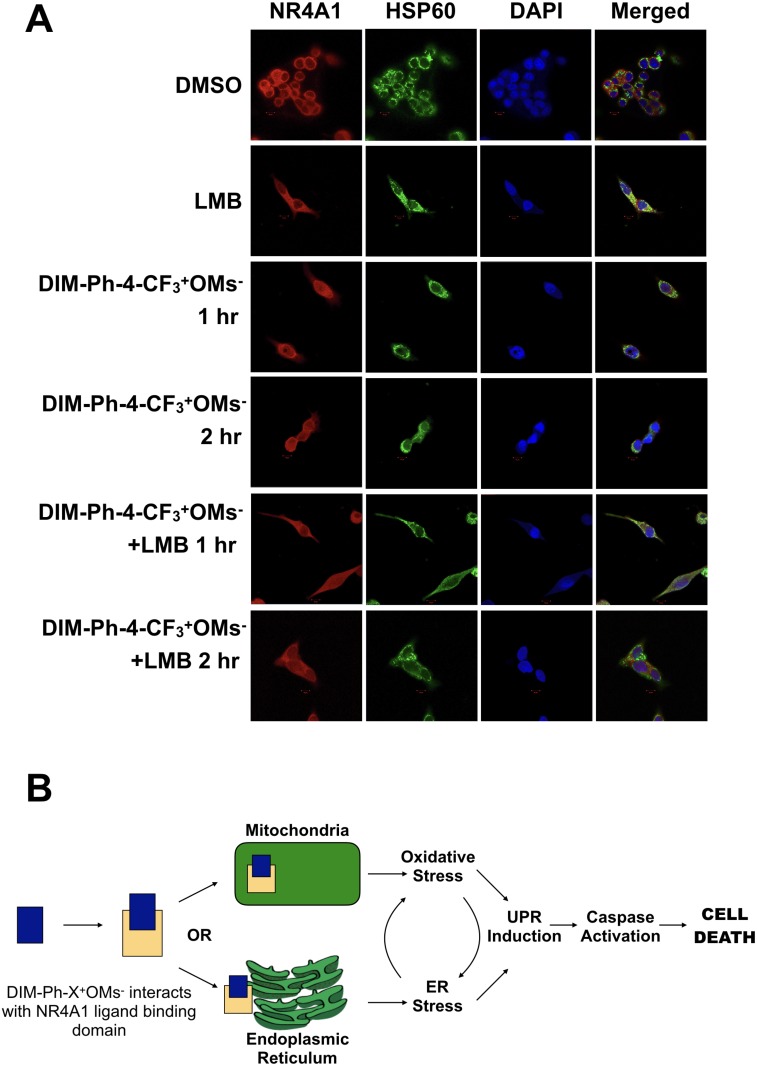
Effect of DIM-Ph-4-CF_3_^+^ OMs^–^ on the localization of NR4A1 (**A**) LNCaP-SKP2 cells were treated with 10 μM DIM-Ph-4-CF_3_^+^OMs^-^ for either 1 or 2 hours. Where indicated, cells were pre-treated with Leptomycin B (50 nM) for 1 hour prior to DIM-Ph-4-CF_3_^+^OMs^-^ treatment. The localization of NR4A1 and mitochondria (HSP60) was then analyzed by immunofluorescence staining as described in the methods. (**B**) Binding of DIM-Ph-4-Xs to NR4A1 may induce specific apoptotic pathways, a potential mechanism of action of DIM-Ph-CF_3_^+^OMs^-^. See discussion for description.

Upon addition of DIM-Ph-4-CF_3_^+^OMs^-^ to LNCaP-SKP2 cells for 1 or 2 hours, the localization pattern of NR4A1 did not change and the receptor was largely retained in the extranuclear compartment (Figure 6A). Similarly, blocking nuclear export with Leptomycin B did not affect the localization of NR4A1 (Figure 6A), suggesting limited nuclear-cytoplasmic dynamics of the receptor within the time frame tested. Interestingly, there was partial overlap between the staining patterns of NR4A1 and the mitochondrial chaperone HSP60 (Figure 6A). In conclusion, these studies did not reveal any evidence of rapid nuclear-cytoplasmic shuttling of NR4A1 either before or after addition of DIM-Ph-4-CF_3_^+^OMs^-^. The data is therefore consistent with an extranuclear mechanism-of-action of DIM-Ph-4-CF_3_^+^OMs^-^-stimulated NR4A1, perhaps through interaction with mitochondria and/or the ER.

## DISCUSSION

In the present work, we abbreviated names of di(indolyl)methyl-4-X-substituted benzenes as DIM-Ph-4-Xs, rather than using other reported names to facilitate comparisons and simplify naming oxidation products as methyleneindoles - (I(Ph-4-X)MIs) - and their salts as DIM-Ph-4-X^+^ OMs^–^s. After air exposure, the DIM-Ph-Xs, which we originally isolated as white powders, became reddish. While air oxidation of DIM-Ph-Xs produced low yields of DIM-Ph-4-X^+^ OMs^–^s, use of pure oxygen raised yields (Figure 1). Yields were further increased using DDQ oxidation and transferring the MsOH addition step to isolated I(Ph-4-X)MIs (X = CF_3_ and CO_2_Me).

Our biological results indicate that DIM-Ph-4-X^+^ OMs^–^s (X = CF_3_, CO_2_Me, Cl and OMe) are considerably more potent inhibitors of cancer cell proliferation than DIM-4-Ph-Xs. Analogs with electron-withdrawing groups (X = CF_3_, CO_2_Me and Cl) were more active than those with an electron-donating group. Electronic effects (σ_p_ values) for the 4-X groups (CF_3_, 0.54; CO_2_Me, 0.45; Cl, 0.23; and OMe, –0.27) of active DIM-Ph-4-X^+^ OMs^–^s appear to correlate inversely with their IC_50_ values (Tables 1–3). DIM-Ph-4-CO_2_H^+^ OMs^–^ inactivity suggests that its electron-withdrawing CO_2_H (σ_p_ value 0.45) was deprotonated to the weak electron donor CO_2_^–^ (σ_p_ value 0.0) or that its uptake was prevented. Growth inhibition by DIM-Ph-4-X^+^ OMs^–^s was independent of 4-X group volume. For example, Ph-4-CF_3_^+^ OMs^–^ with an intermediate volume CF_3_ was more active than Ph-4-OMe^+^ OMs^–^ having a 14% smaller OMe and just as active as Ph-4-CO_2_Me ^+^ OMs^–^ having a 33% larger CO_2_Me.

With one exception (DIM-Ph-4-CF_3_), DIM-Ph-4-X IC_50_ values were not reached after 72-h treatments at 2.0 μM. A report showed DIM-Ph-4-X activities at higher concentrations (5–20 μM), which we confirmed for DIM-Ph-4-CF_3_ (IC_50_ approx. 8.0 μM against LNCaP cells after 72 h). Our results suggest that the DIM-Ph-4-Xs could function as prodrugs *in vivo* by being converted to more active I(Ph-4-X)MIs or their salts by oxidation, as we demonstrated for DIM-Ph-4-Xs (X = CF_3_ and CO_2_Me) (Figure 1).

To understand how the Ph-4-X ^+^ OMs^–^s inhibited cell viability and demonstrate their higher potency, we focused on DIM-Ph-4-X activities reported by Safe and colleagues. These authors demonstrated inhibition of cancer cell proliferation and function as NR ligands with DIM-Ph-CF_3_ being an NR4A1 [14] and PPARγ [15–17, 20] agonist, and DIM-Ph-4-OMe a selective NR4A1 agonist [14]. However, the DIM-Ph-4-X signaling pathways for cancer cell inhibition were observed to be both dependent and independent of NR4A1 and PPARγ interaction [14, 31].

Using NMR spectroscopy and DSC, we found that DIM-Ph-4-CF_3_^+^ OMs^–^ interacted with the NR4A1 LBD, whereas interaction with DIM-Ph-4-CF_3_ was undetectable by NMR and very low by DSC under our conditions. Subsequent ligand docking studies to NR4A1 LBD allosteric sites provided detailed structural hypotheses for ligand-receptor interaction that can be explored by specific mutations in future studies. Our observation that DIM-Ph-4-X^+^ OMs^–^-mediated apoptosis of LNCaP-SKP2 cells was abolished upon knockdown of NR4A1 strongly suggests that, at least in this cell line, compound-induced apoptosis depends on NR4A1 and may thus be mediated by receptor-ligand interaction.

Several lines of evidence suggest that DIM-Ph-4-CF_3_^+^ OMs^–^ exerts its pro-apoptotic activity primarily through extranuclear NR4A1: (i.) At doses tolerated by cells, neither DIM-Ph-4-CF_3_ (2 μM) nor DIM-Ph-4-CF_3_^+^ OMs^–^ (0.1 μM) affected NR4A1-dependent transcriptional reporter activity more than 1.3 – 1.6-fold (data not shown), suggesting that these compounds are not potent activators of genomic NR4A1 functions. (ii.) We found in three different cancer cell lines that NR4A1 is substantially localized outside of the nucleus and does not appear to undergo major relocalization upon exposure of cells to DIM-Ph-4-CF_3_^+^ OMs^–^. (iii.) At cytotoxic doses, DIM-Ph-4-CF_3_^+^ OMs^–^ is a potent inducer of the unfolded protein response, which is initiated in the endoplasmic reticulum.

In summary, our findings are consistent with a first framework of the mechanism-of-action of DIM-Ph-4-Xs as anti-cancer agents (Figure 6B): The compounds interact with the LBD of NR4A1. The NR4A1 bound to DIM-Ph-4-Xs may induce specific apoptotic pathways. Presumably due to ligand-induced targeting to the ER [11, 31], NR4A1 might trigger the UPR which signals downstream cell death pathways via IRE1α signaling [32]. UPR induction leads to an increase in caspase 3 and 7 activity, which mediates cell death. Oxidative stress is a well-established UPR inducer and could be involved in this mechanism as well [33]. Consistent with this possibility is the observation that NR4A1 localizes to mitochondria in a ligand-dependent manner [6, 7]. Cancer cell selectivity may then arise from the higher tolerance of normal cells to both oxidative and ER stress relative to cancer cells with their stress defense already being stretched to the limit [34].

## MATERIALS AND METHODS

### Synthetic chemistry

#### Oxidation of DIM-Ph-4-Xs to DIM-Ph-4-X^+^ OMs^–^s (X = CF_3_, CO_2_Me, Cl and OMe)

Procedures are outlined in Figure 1. Supplementary data (Supplementary Data) provide reaction conditions for DIM-Ph-4-X^+^ OMs^–^s and DIM-Ph-4-Xs and chemical characterization data.

#### Oxidation of DIM-Ph-4-Xs (X = CF_3_ and CO_2_Me) to I(Ph-4-X)MIs (X = CF_3_ and CO_2_Me)

A solution of DIM-Ph-4-X and 2,3-dichloro-5,6-dicyano-p-benzoquinone (DDQ) in acetonitrile (MeCN) was stirred for 2–3 h and filtered (Figure 1). The dark-red solid was washed (EtOAc or MeCN, and Et_2_O). Chromatography gave I(Ph-4-X)MI in >70% yield. Supplementary Data provides reaction conditions and chemical characterization data.

### Computational procedures

The supplement describes methods for calculating X group volume and small-molecule docking to NR4A1 LBD crystallographic structures (PDB 2QW4 [22] and 3V3Q [13]).

### Ligand binding to NR4A1

#### ^19^F NMR spectroscopy

NR4A1 (TR3) LBD (residues 467−598) and its C-terminal deletion mutant NR4A1(WII4) (residues 1–467) were expressed in *Escherichia coli* Bl21 as N-terminal His_6_ constructs and purified by chromatography (His60 Ni Superflow nickel-tethered resin, Clontech) and dialysis. ^19^F NMR spectra on samples cooled to 11° C were recorded using a 500-MHz Advance NMR spectrometer (Bruker) having a fluorine probe and using 128 transients, a 14,124-Hz sweep width and a 3-sec repetition time. Samples contained 50.0 μM DIM-Ph-4-CF_3_^+^ OMs^–^ or DIM-Ph-4-CF_3_ alone in buffer (20 mM Tris·HCl, pH 8.0, and 400 mM NaCl) in 86.4% D_2_O and 2.0% DMSO-d_6_ or with added 5.0 μM NR4A1 LBD or NR4A1(WII4) mutant protein.

#### Differential scanning calorimetry

Methods are detailed in the supplement.

### Antibodies and reagents

Antibodies (Abs) and vendors were: Rabbit anti-IRE1α monoclonal antibody (MAb), rabbit anti-BiP MAb, rabbit anti-phospho-eIF2α(Ser 51) Ab and rabbit NR4A1 XP**^®^** MAb (Cell Signaling Technology); mouse anti-actin MAb (MP Biomedicals); HRP-linked donkey anti-mouse IgG and HRP-linked donkey anti-rabbit IgG (Jackson ImmunoResearch Laboratories); goat anti-rabbit IgG–Alexa Fluor**^®^** 594 conjugate (Life Technologies); rabbit polyclonal anti-PARP Ab (Santa Cruz Biotech); and horseradish peroxidase-conjugated secondary anti-rabbit IgG and anti-β-actin Ab (Sigma). 4´,6-Diamidino-2-phenylindole (DAPI) was from Sigma.

### Tissue culture

Characteristics of human cancer and leukemia cell lines are described and cited in Supplementary Data. HCT-116 colon cancer cells were cultured in McCoy's 5A medium (Invitrogen), MCF-7 breast cancer cells in DMEM containing glucose (4.5 mg/mL) (Invitrogen) and 2.0 mM glutamine, and LAPC-4 prostate cancer cells in IMDM (Invitrogen). MDA-MB-231 breast cancer and LNCaP, 22Rv1 and PC-3 prostate cancer cells were cultured in RPMI-1640 (CellGro, Mediatech) supplemented with 5% penicillin–streptomycin (Omega Scientific). PC-3 medium also contained 4.5 mg/mL glucose. Unless noted, cells were grown and evaluated in media containing 10% FBS (Hyclone). HCT-116, MCF-7, MDA-MB-231, LAPC-4 and 22Rv1 (all 10 × 10^3^/well) and LNCaP and PC-3 cells (5 × 10^3^/well) were plated into 96-well plates, allowed to attach for 24 h at 37° C before 72-h treatments.

CV-1 cells were maintained in DMEM with 10% FBS and grown to 60–80% confluency before transfection with plasmids using Lipofectamine 2000 (Invitrogen). After 16 h, cells were treated with 0.1 μM compound for 2 h before immunoblotting [35]. COS-7 cells cultured in 10-cm dishes containing DMEM, 10% FBS and 1% antibiotic/antimycotic cocktail until 80% confluency were transfected with 6 μg CMX-empty or CMX-PPARγ, 3 μg PPRE-Luc and 1 μg TK-Renilla plasmids using jetPRIME**^®^** (Polyplus Transfection). At 24-h post-transfection, cells (8 × 10^4^) were seeded into 96-well plates. After 10 h, cells were treated with compound for 14 h.

Dr. Michael Andreeff (M.D. Anderson Cancer Center, Houston, TX) provided KG-1, MOLM-13, OCI-AML-2, OCI-AML-3 and THP-1 AML cell lines. K562 CML and MOLT4 T-ALL lines were from ATCC**^®^**. Supplementary Table 3 lists AML cell characteristics. Cells were cultured for 24 h in RPMI-1640, 10% FBS and 1% penicillin–streptomycin cocktail before treatment for 24 h.

### Viability assays

#### MTT assay

Cancer cell lines were treated with 0.125–2.0 μM compound in DMSO or DMSO alone (0.2% final concentration) for 72 h. Viability was assessed by MTT assay (ATCC**^®^**) using the manufacturer's protocol. Results are averages of triplicates ± standard deviations (SDs). IC_50_ values were calculated by interpolation of best-fit concentration–response curves.

#### ATP assay

Leukemia suspensions (2.5 × 10^4^ cells/100 μL medium/well) were added to 96-well white-bottomed Greiner plates (E&K Scientific Products) containing medium plus 10% FBS and DMSO alone or with compound in DMSO (0.125% final concentration, which did not affect cell growth). After 24 h, ATP levels were determined using CellTiter-Glo**^®^** Luminescent Cell Viability Reagent (50 μL/well; Promega) followed 10 min later by measuring light emission (FlexStation 3 Microplate Reader, Molecular Devices). Experiments were in triplicate (mean ± SD) with viabilities calculated using maximum luminescence intensity for each line in the DMSO control (100% value).

### DAPI assay

Effects on proliferation of MMTV-Wnt1 murine mammary cancer stem cells derived from mammospheres were determined as described [29]. Disassociated cells were plated onto gelatin-coated 384-well plates, incubated for 24 h and then treated with compound in DMSO (0.1% final concentration) or DMSO alone for 72 h. Cells were fixed and stained (DAPI). Cell numbers were determined on triplicates by analyzing nine images acquired using the IC100 automatic focusing imaging system and CyteSeer image analysis software (Vala Sciences) at 358-nm excitation and 461-nm emission. Results are means ± SD of triplicates.

#### Clonogenicity assay

LNCaP-SKP2 cells were plated at equal densities (5 × 10^4^ cells/dish) in 10 cm dishes and maintained for 3 weeks. Cells were treated with DMSO or 2 μM DIM-Ph-4-CF_3_^+^OMs^-^ during the 3-week culture period. The media was changed every 7 days and fresh compound was added with fresh media. The colonies were stained with 1% crystal violet in 10% ethanol for 30 minutes and the numbers of colonies were counted using CFU Scope v1.4 software by mediXgraph.

### Immunoblotting

Cells were incubated in lysis buffer (50 mM Tris·HCl, pH 7.9, 150 mM NaCl, 1 mM EDTA, 1% Triton X-100 and protease inhibitor cocktail (Roche Applied Science)) on ice for 10 min. After a 15-min centrifugation (15000 × *g)*, lysates were boiled in SDS sample buffer. Proteins were resolved on 10% SDS–PAGE gels and transferred to nitrocellulose membranes, which were blocked by 5% milk in TBST (10 mM Tris·HCl, pH 8.0, 150 mM NaCl and 0.05% Tween 20) for 30 min and then incubated with TBST containing the Ab for 2 h. Membranes were washed (TBST 3X) and incubated in TBST containing horseradish peroxide-linked anti-IgG for 2 h. After washing (TBST 3X), immunoreactive products were detected by chemiluminescence using enhanced chemiluminescence (Amersham).

## Cell apoptosis assays

### PARP cleavage

HCT-116 cells grown in RPMI-1640 medium with 10% FBS were seeded into 6-well plates, allowed to attach (12 h) and treated with 1.0 μM DIM-Ph-4-CF_3_^+^ OMs^–^ for 6 h. Cells were centrifuged and lysed (50 mM Tris·HCl, pH 7.9, 150 mM NaCl, 1 mM EDTA, 1% Triton X-100 and protease inhibitor cocktail (Roche Applied Sciences)). Lysates were loaded onto 12% SDS–PAGE gels. Separated proteins were transferred to nitrocellulose membranes, which were blocked using 5% nonfat milk in TBST buffer (20 mM Tris·HCl, pH 8.0, 150 mM NaCl and 0.05% Tween 20) for 30 min, washed (TBST 2X), probed with rabbit polyclonal anti-PARP Ab overnight at 4° C and then treated with horseradish peroxidase-conjugated anti-rabbit IgG for 2 h. Blots were developed using enhanced chemoluminescence (GE Healthcare) and then reprobed with anti-β-actin Ab to confirm equivalent protein loading.

## DNA fragmentation and caspase activation

Cytoplasmic histone-associated DNA fragmentation was measured using the cell death detection ELISA kit (Roche Applied Sciences) and caspases-3/7 activities using the Caspase-Glo 3/7 assay kit (Promega) according to manufacturers’ instructions.

## Cell morphology

Apoptosis of prostate cancer cells treated with compounds for 24 h was evaluated qualitatively by visualization under a Nikon Type 120 inverted microscope at 10× magnification.

## UPR detection

LNCaP (3 × 10^5^) cells in medium were treated for 24 h with DMSO alone (0.02% final concentration) or compound in DMSO, then collected (1000 rpm) and suspended in 1.0 mL PBS. Each suspension was divided. One half was spun to collect cells, which were lysed at 4 °C for 15 min using lysis buffer (25 mM Tris·HCl, pH 7.4, 150 mM NaCl and 0.5% Triton X-100). Protein levels in lysate aliquots were determined using the Bradford assay and a BSA standard. The remaining lysate was subjected to 4–20% SDS–PAGE gel electrophoresis. Gel bands were transferred to nitrocellulose membranes, which were analysed by immunoblotting for IRE1α, BiP, p-eIF2α and β-actin as described [36]. Films of immunoblots were scanned, and protein bands quantified using Image J software (http://rsbweb.nih.gov/ij/). The second half of the cell pellet was used to determine cytoplasmic histone-associated DNA fragment levels by the manufacturer's instructions.

## XBP1 splicing

Total RNA was isolated from LNCaP-SKP2 cells using the Qiagen RNeasy kit. RT-PCR was performed using the Invitrogen OneStep RT-PCR kit. The following primers were used to detect XBP1 splicing:

**Table:** 

	Forward 5ʹ → 3ʹ	Reverse 5ʹ → 3ʹ	Reference
XBP1	CCTTGTAGTTGAGAACCAGG	GGGGCTTGGTATATATGTGG	[36]

## siRNA knockdown and immunofluorescence staining

### siRNA knockdown

Cells (2 × 10^4^ cells) were plated on glass coverslips one day prior to transfection. 10–20 nM of siRNA was transfected using Lipofectamine 2000 (Invitrogen) according to the manufacturer's instructions. Briefly, siRNAs were added to the Lipofectamine 2000 reagent prediluted in OptiMEM media (1:100) and incubated for 45 min at room temperature. The mixture was added to the cells followed by incubation for 48 hrs. Un-transfected cells as well as cells transfected with nontargeting siRNA (Signal Silence, Cell Signaling) were used as controls. siRNA targeting Nur77 (sc-36109) was purchased from Santa Cruz Biotechnology.

### Immunofluorescence staining

Cells were cultured overnight on glass coverslips and then treated with the reagents described above. Cells were fixed for 5 min with 4% paraformaldehyde, permeabilized with 0.2% Triton X-100, 0.04% SDS in PBS for 5 min, and then incubated for 30 min in a 3% bovine serum albumin (BSA)/PBS blocking solution. NR4A1/Nur77 was detected with a 1:100 diluted rabbit NR4A1 XP^®^ MAb (Cell Signaling Technology) followed with 1:100 diluted goat anti-rabbit AlexaFluor 594-conjugated secondary antibody (Invitrogen). To identify the mitochondria, a 1:200 diluted mouse monoclonal Hsp60 antibody was used (Calbiochem), followed by 1:100 diluted goat anti-mouse AlexaFluor 488-conjugated secondary antibody (Abcam). All secondary antibodies were incubated for 1 hr at 37° C. To visualize nuclei, cells were stained with 0.3 μg/ml DAPI for 5 min. Finally, immunofluorescence staining was assessed using a fluorescence microscope (Zeiss LSM710 NLO Multiphoton microscope) equipped with image acquisition system. Images were analyzed using Zeiss ZenBlack software.

## Generation of stable LNCaP-SKP2 cell line

5 × 10^6^ parental LNCaP prostate cancer cells were seeded in 10 cm dishes in 10 mL of complete RPMI-1640 medium with 10% FBS 24 hours prior to transfection. On the day of transfection, cells were washed with 5 mL Opti-MEM once and then incubated with fresh Opti-MEM for 45 minutes at 37° C. 20 ug of plasmid was diluted in Opti-MEM for a final volume of 250 ul. 50 ul of Lipofectamine 2000 was then added to plasmids and incubated for 45 minutes. Cells were then transfected with pcDNA3.1 SKP2 plasmid or pcDNA3.1 control plasmid and incubated for 24 hours. Stable cell lines were selected in 700 mg/mL G418 for 7 days. Single cells were selected and grown in 96 well plates to establish monoclonal cell lines. Monoclonal cell lines were expanded and validated for SKP2 overexpression via Western blotting (Supplementary Figure 7).

## LNCaP-SKP2 xenograft studies

Animal experiments were performed in accordance with procedures approved by the institutional animal care committee (IACUC) of Sanford Burnham Prebys Medical Discovery Institute, La Jolla, CA. NOD/SCID mice (8–10 weeks old) were procured from Jackson Laboratories, Inc., housed under pathogen-free conditions, and maintained on a 12 h libitum/ 12 h dark cycle, with food and water supplied ad libidum. LNCaP-SKP2 cells (3 × 10^6^ cells as a 50% suspension in Matrigel, BD Biosciences, San Jose, CA) in a final volume of 0.2 ml were injected subcutaneously in the flank region of the animals. Tumor sizes were measured every day until they reached ~150–200 mm^3^. Mice were then injected intraperitoneally (i.p.) with vehicle (80% 1 x PBS, 100% ethanol and 10% Cremaphor EL) or DIM-Ph-4-CF_3_^+^OMs^-^ (15 mg/kg) daily for 18 days. Body weights and tumor sizes were measured daily. Tumor volume was calculated using the equation: volume = length × width × depth × 0.5 mm^3^. At the end of the experiment, animals were sacrificed.

## Immunohistochemistry

LNCaP-SKP2 xenografts were grown in mice until tumors were 200–300 mm^3^ in size. Mice were then injected with either vehicle or 10 mg/kg DIM-Ph-4-CF_3_^+^OMs^-^ every 12 hrs for 24 hrs. Tumors were then harvested and fixed in 4% paraformaldehyde for 24 hrs. Sections of tissue were stained with 1:200 of rabbit anti-BiP antibody (CST) for detection of unfolded protein response (UPR) induction.

## H score calculation

H scores were obtained by the following formula: (3 x percentage of strongly staining nuclei) + (2 x percentage of moderately staining nuclei) + (percentage of weakly staining nuclei), giving a range from 0 to 300.

## SUPPLEMENTARY MATERIALS FIGURES AND TABLES




