# Supplementary material for: Oxidized analogs of Di(1H-indol-3-yl)methyl-4-substituted benzenes are NR4A1-dependent UPR inducers with potent and safe anti-cancer activity
Source: Oncotarget. 2018 May 18;9(38):25057–74. doi: 10.18632/oncotarget.25285 (PMC5982742; doi:10.18632/oncotarget.25285)
Supplement: Supplementary file 1 [file oncotarget-09-25057-s001.pdf]

Oxidized analogs of Di(1*H*-indol-3-yl)methyl-4-substituted benzenes are NR4A1-dependent UPR inducers with potent and safe anti-cancer activity

SUPPLEMENTARY MATERIALS

Supplementary Table 1: Monitoring oxidation using HPLC

	Peak area[a] (%)			
**Reaction time (h)**	**DIM-Ph-4-CF_3**	**DIM-Ph-4-CF_3^+ OMs^-**	**DIM-Ph-4-CO_2Me**	**DIM-Ph-4-CO_2Me^+ OMs^-**
0	98	0	96	0.9
21	49	39	51	34
46	34	49	39	43

[a]Determined by HPLC using the same conditions as those for compound purity analyses (see below). After 46 h, the reactions were worked up, and DIM-Ph-4-X^+ OMs^-s were isolated in 36% and 32% yields, respectively.

Supplementary Table 2: HPLC analytical data for analogues using the A/B solvent system

**DIM-Ph-4-X^+ OMs^- X**	**HPLC analysis**	
	**t_R (min)**	**peak area (%)**
CF_3	14.98	100
CO_2Me	13.70	98
Cl	15.08	96
OMe	14.07	100
CO_2H	12.66	96
**I(Ph-4-X)MI X**		
CF_3	14.91	100
CO_2Me	13.74	100

Supplementary Table 3: Characteristics of leukemia cell lines --KG-1, MOLM-13, OCI-AML-2, OCI-AML-3 and THP-1 AML, K562 CML and MOLT-4 T-ALL[a,b]. See Supplementary_Table _3

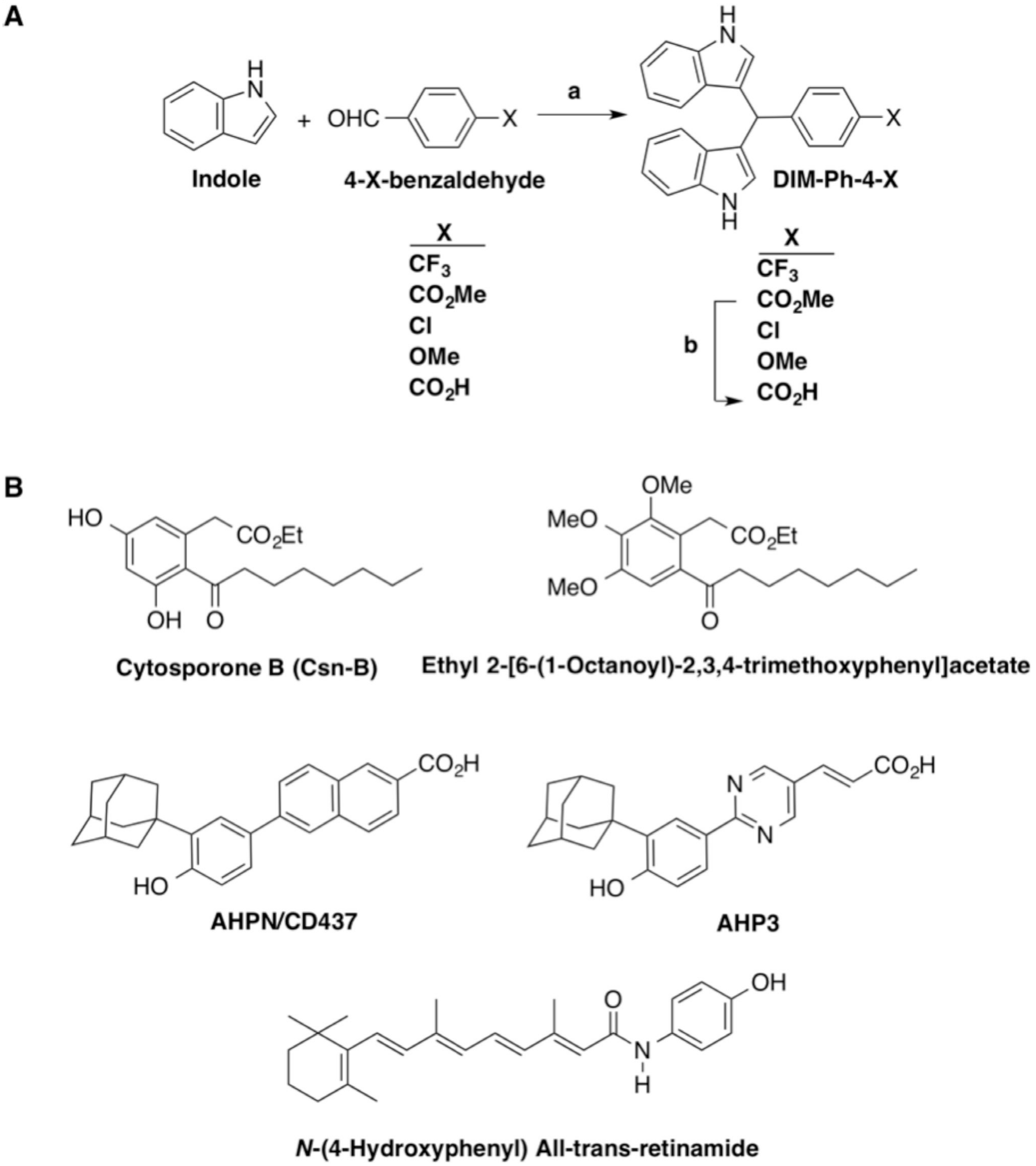

Supplementary Figure 1: Synthesis of DIM-Ph-4-Xs and structures of apoptosis-inducing agents. (**A**) Reagents and conditions: (a) $CeCl_3 \cdot 7H_2O$/NaI on silica gel, CH_3CN. (b) 5 M aq NaOH, MeOH, reflux; H_3O^+. (**B**) Chemical structures of NR4A1 agonist and apoptosis-inducer cytosporone B, NR4A1 antagonist ethyl 2-[6-(1-octanoyl)-2,3,4-trimethoxyphenyl]acetate (transcriptionally inactive), 6-[3-(1-adamantyl)-4-hydroxyphenyl]-2-naphthalenecarboxylic acid (AHPN/CD437), (*E*)-3-{2-[(1-adamantyl)-4-hydroxyphenyl]-1,3-pyrimid-5-yl}-2-propenoic acid (AHP3) and N-(4-hydroxyphenyl) all-trans-retinamide.

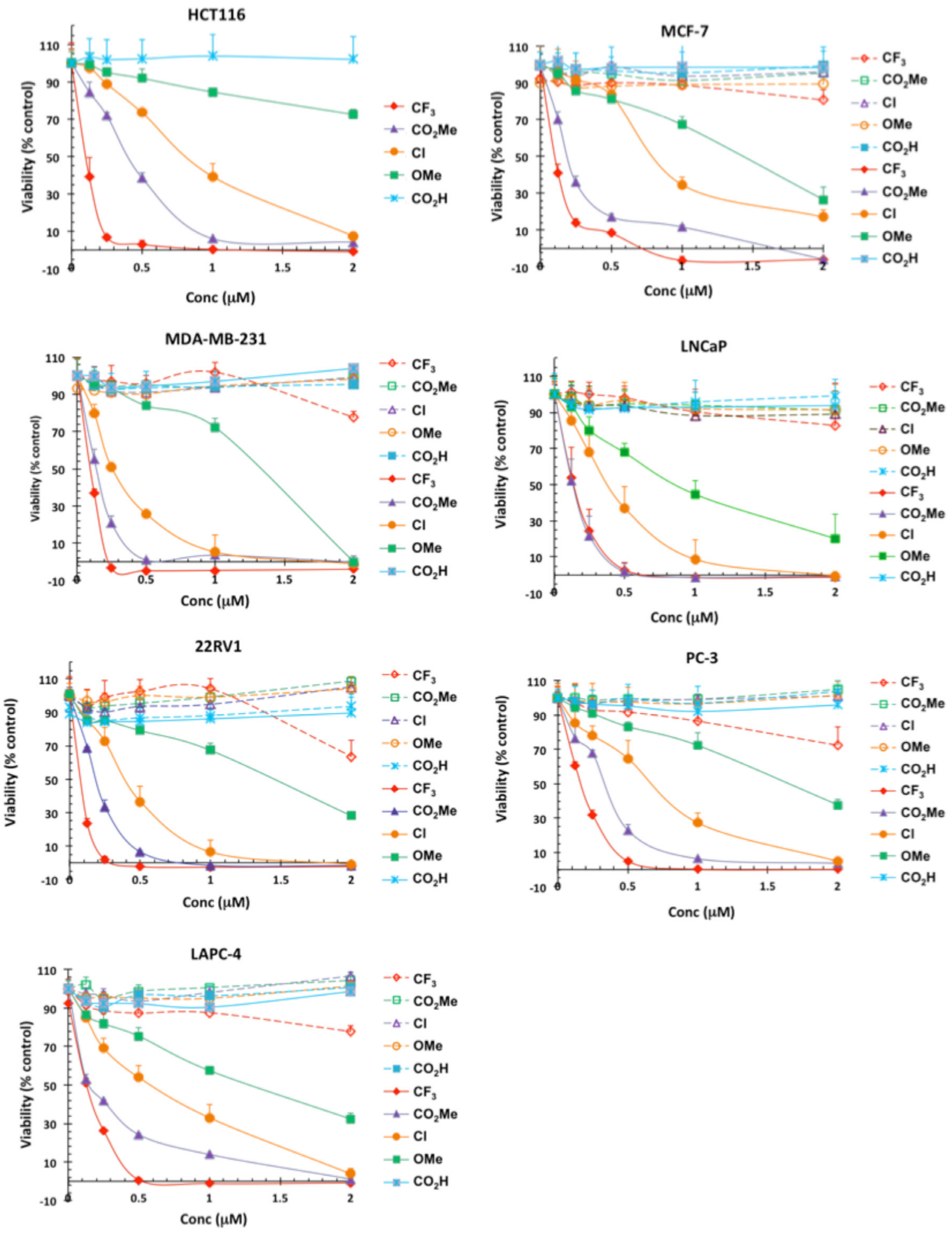

Supplementary Figure 2: Effects of DIM-Ph-4-Xs and the mesylate salts DIM-Ph-4-X$^+$ OMs$^-$s on viability of cancer cell lines. Cancer cell lines having various metastatic potentials—HCT-116 colon, MCF-7 and MDA-MB-231 breast, and LNCaP, 22RV1, PC-3 and LAPC-4 prostate cancer—as measured by the MTT cell viability assay as described in the Methods. Cells were plated in 96-well plates, allowed to attach for 24 h and then treated with each compound at increasing concentrations (0.125 to 2.0 mM) in DMSO or DMSO alone (0.2% final concentration) for 72 h. Viability of treated cells relative to the vehicle-treated control is expressed as means of quadruplicates ± SD of the ratios of treated to control cells × 100. Concentration–viability response curves are represented as dashed lines and open symbols for the DIM-Ph-4-Xs (parent compounds) and as solid lines and closed symbols for the DIM-Ph-4-X$^+$OMs$^-$s (oxidized compounds). Individual compounds are designated by their X substituent at the phenyl ring 4-position (X = CF_3, CO_2Me, Cl, OMe and CO_2H). IC_{50} values listed in Tables 1 and 2 were determined from these concentration–viability response curves.

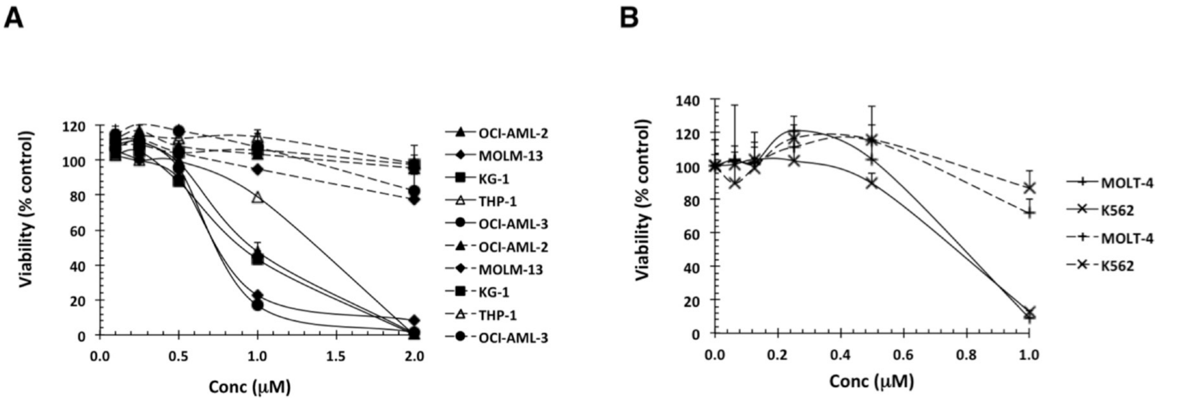

Supplementary Figure 3: Effects of DIM-Ph-4-CF_3 and DIM-Ph-4-CF_3^+ OMs^- on leukemia cell viability as measured by changes in ATP levels. Cells were grown for 24 h in RPMI 1640 with 10% FBS and 1% penicillin-streptomycin at 37° C and then treated for 24 h with compounds at the indicated concentrations in DMSO (0.125% final concentration) or DMSO alone. Data points represent of the means of triplicates ± SD of the ratios of the viabilities of the treated to vehicle control cells ´ 100. Concentration–viability response curves for DIM-Ph-4-CF_3 and DIM-Ph-4-CF_3^+ OMs^- on (**A**) OCI-AML-2 (▲), MOLM-13 (◆), KG-1 (■), THP-1 (D) and OCI-AML-3 (●) AML cell lines and on (**B**) MOLT-4 T-ALL (+) and K562 CML (×) cell lines are represented by dashed lines and solid lines, respectively. IC_{50} values were determined by interpolation of the concentration–viability curves and are listed in Table 3.

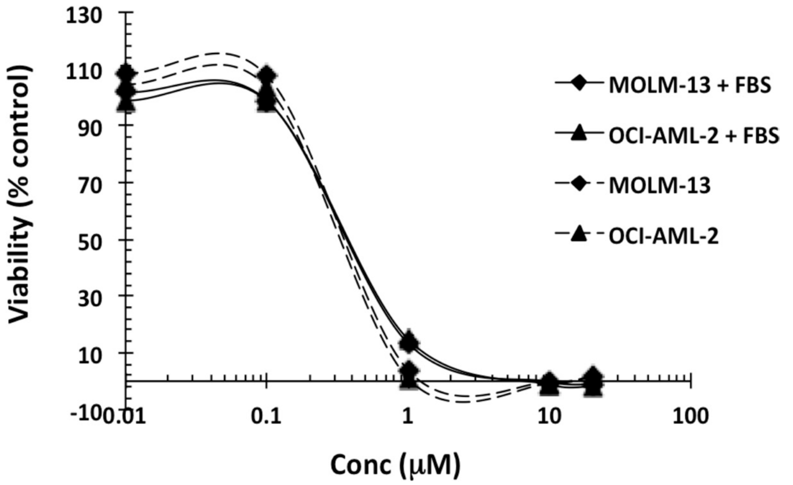

Supplementary Figure 4: Fetal bovine serum (10%) in growth medium does not have a major impact on AML growth inhibition by DIM-Ph-4-CF_3^+ OMs^-. MOLM-13 and OCI-AML-2 AML cells were grown in RPMI 1640 medium with and without 10% fetal bovine serum (FBS) and then treated for 24 h with 0.01, 0.1, 1.0 and 10.0 μM in DMSO (0.125% final concentration) or DMSO alone. Inhibition of cell viability was determined by measuring the decline in ATP levels as described in the Methods. Viabilities of treated cells relative to those of the vehicle-treated control cells were expressed as the ratios of means of triplicates ± SD × 100. Solid lines represent OCI-AML-2 (AML-2) (▲) and MOLM-13 (◆) cells grown in the presence of FBS and dashed lines those grown in its absence. IC_{50} values were determined from interpolation of the concentration–viability curves and are listed in Table 4.

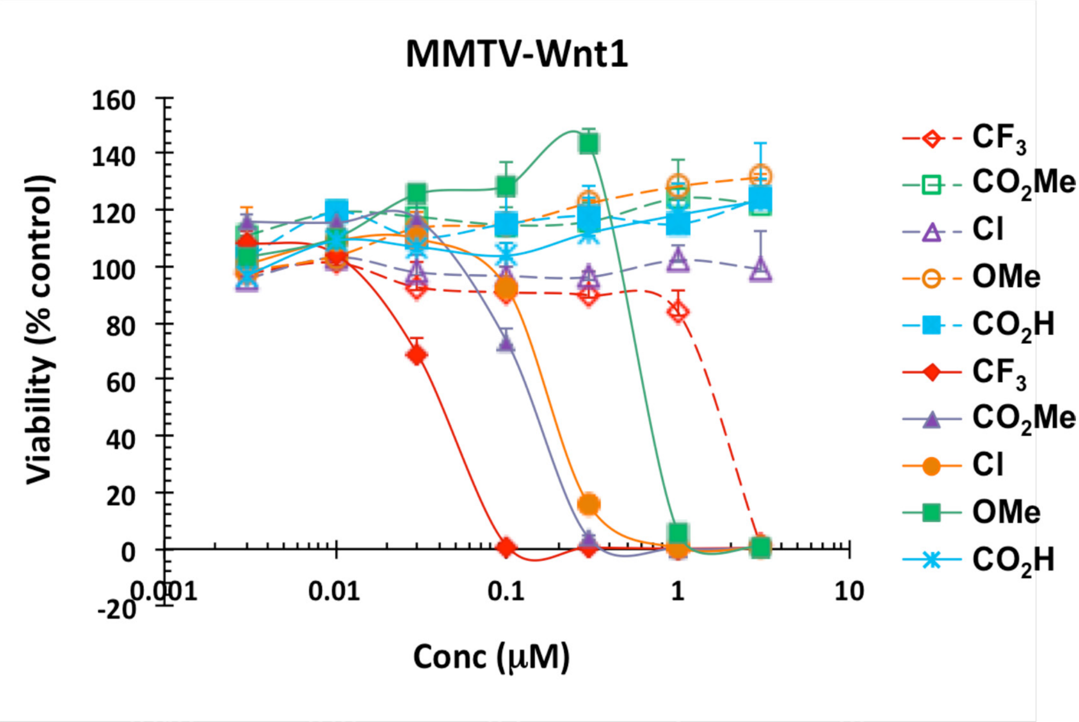

Supplementary Figure 5: Effects of DIM-Ph-4-Xs and DIM-Ph-4-X$^+$ OMs$^-$s on MMTV-Wnt1 murine mammary cancer stem cell viability. Cells were treated for 72 h with each compound at the indicated concentrations in DMSO or with DMSO alone (control, 0.1% final concentration) before the number of viable treated cells relative to vehicle-treated control cells was determined as described in the Methods. Data points represent the means of triplicates ± SD of the ratios of treated to control cell levels ´ 100. Concentration–viability response curves for DIM-Ph-4-Xs are represented by dashed lines and open symbols and those for DIM-Ph-4-X$^+$ OMs$^-$s by solid lines and solid symbols. Individual compounds are designated by their X substituent (X = CF_3, CO_2Me, Cl, OMe and CO_2H). IC_{50} values for 50% viability reduction listed in Table 1 were determined by interpolation of these curves.

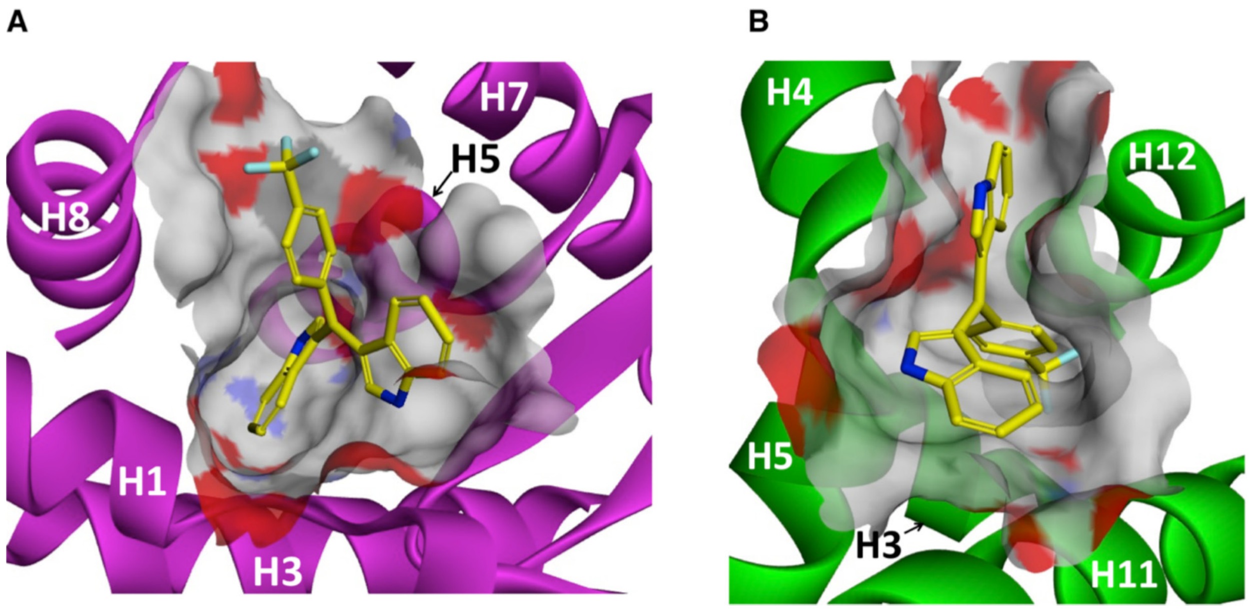

Supplementary Figure 6: The cation (DIM-Ph-4-CF_3^+) of DIM-Ph-4-CF_3^+ OMs^- docks to the two allosteric binding sites of the NR4A1 LBD protein structure (PDB 3V3Q). (**A**) Low-energy conformation of DIM-Ph-4-CF_3^+ docked to allosteric site 1 with adjacent site (LBP) helices H1, H3, H5, H7 and H8 shown in magenta. (**B**) Low-energy conformation of DIM-Ph-4-CF_3^+ docked to allosteric site 2 with adjacent site helices H3, H4, H5, H11 and H12 shown in green. The allosteric site surfaces around DIM-Ph-4-CF_3^+ together with key helices in sites 1 and 2 within 8 Å are shown. The pose for DIM-Ph-4-CF_3^+ is shown in stick format with C atoms in yellow, N in blue, and F in light-blue. Allosteric site surface N atoms are colored in blue; Os in red. H atoms were omitted for clarity. Docking studies employed GOLD software in the Discovery Studio 3.5 package.

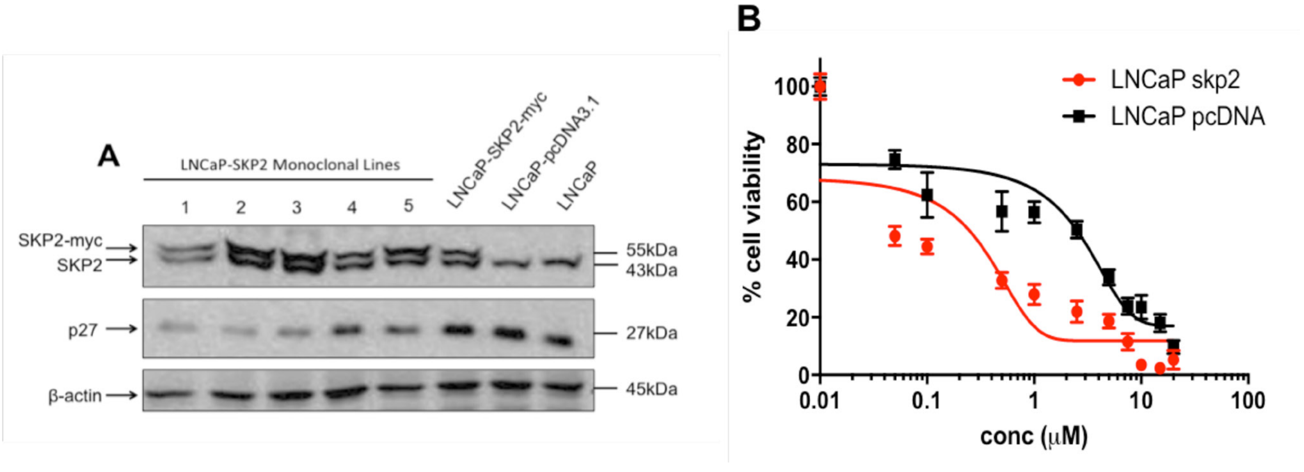

Supplementary Figure 7: LNCaP-SKP2 cells maintain sSupplementary Table KP2 overexpression. (**A**) Each of the five monoclonal LNCaP cell lines single cell clones from SKP2 overexpressing polyclonal lines were tested for stable overexpression of SKP2 by Western blotting. LNCaP parental cells and LNCaP cells transfected with pcDNA3.1 only were used as controls. P27 expression was found downregulated in clone #1-3. Clone #2 was ultimately used for experiments due to the high level of overexpression of SKP2 and the downregulation of p27. (**B**) Stable LNCaP-SKP2 cells were treated with increasing doses of SMIP004-7 for 96 hours. Cell viability assay determined that LNCaP-SKP2 cells are highly sensitive to SMIP004-7 (IC_{50} 0.3 μM) while LNCaP cells stably transfected with empty pcDNA3.1 are less sensitive (IC_{50} of 3.5 μM).

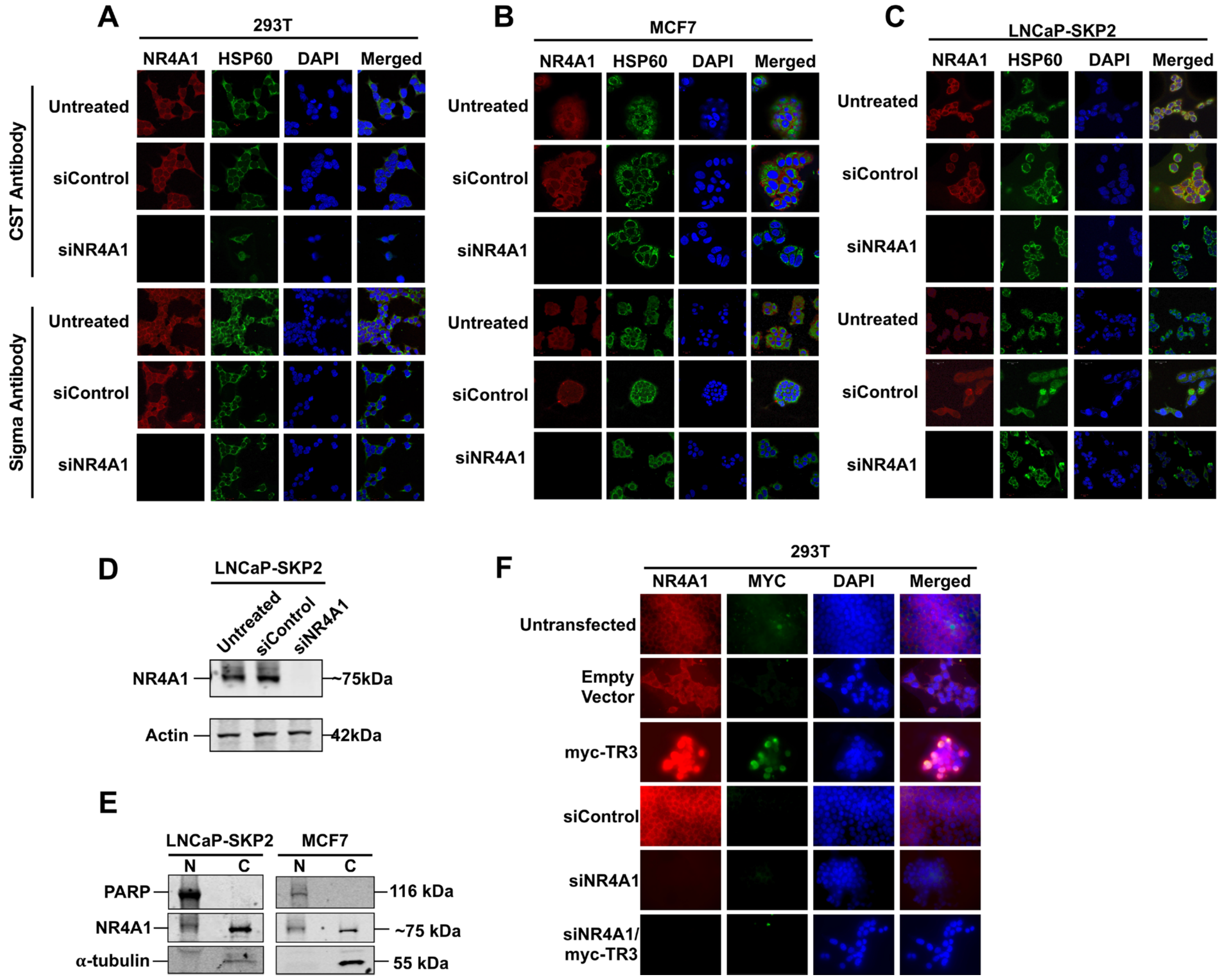

Supplementary Figure 8: Specificity of NR4A1 antibodies and subcellular localization of NR4A1. (**A**) 293T cells were transfected with either siRNA targeting Nur77 (NR4A1) or control siRNA as described in the methods. Knockdown efficiency and specificity was analyzed by immunofluorescence staining with NR4A1 XP® monoclonal antibody (Cell Signaling Technologies) as described in the methods. Immunofluorescence staining demonstrated that the NR4A1 XP® monoclonal antibody specifically recognizes NR4A1. Immunofluorescence results were confirmed with another antibody (NR4A1 polyclonal antibody, Sigma). (**B**) The same tests performed in (A) were done in MCF7 cells. (**C**) The same tests performed in (A) were done in LNCaP-SKP2 cells. (**D**) NR4A1 knockdown efficiency in LNCaP-SKP2 cells was determined by immunoblotting with NR4A1 XP® monoclonal antibody. Actin is shown for reference. (**E**) Nuclear and cytoplasmic fractions from LNCaP-SKP2 and MCF7 cells were analyzed by immunoblotting with the NR4A1 XP® monoclonal antibody. Signals obtained with nuclear marker PARP and cytoplasmic tubulin are shown for reference. (**F**) 293T cells were transfected with either siRNA targeting Nur77 (NR4A1), myc-TR3 plasmid DNA that overexpresses TR3 (NR4A1) or both. NR4A1 XP® monoclonal antibody (Cell Signaling Technologies) specificity was further validated through immunofluorescence staining and analysis.

Monitoring the Oxidation of DIM-Ph-4-Xs (X = CF_3 and CO_2Me) to DIM-Ph-4-X^+ OMs^-s

(X = CF_3 and CO_2Me)

Compound purity analyses

Reversed-phase HPLC analysis of target compounds in Supplementary Table 2 was performed on a Shimadzu 20A HPLC system using elution from a 4.6-mm × 150-mm TITAN C_{18} column (5-μm particle size), with the UV absorbance detection at 254 nm, and the mixed A/B solvent system described below at a flow rate of 1.0 mL/min:

Solvent A: 0.2% H_3PO_4 in H_2O

Solvent B: 0.2% H_3PO_4 in MeOH

Using the solvent gradient 35 min 70% A/30% B to 5% A/95% B (0–20 min) followed by 95% B (20–33 min) and then 5% A/95% B to 70% A/30% B (2-min recycle).

Reported leukemia cell mutations, gene transpositions and over or under-expressed proteins

DIM-Ph-4-CF_3^+ docks to NR4A1 LBD structure 3V3Q

Although the pose in Figure S6A shows that the 4-CF_3-Ph group and benzo portions of the indole rings of the cation are more exposed on the LBD surface, a partial π–π stacking between its phenyl ring and that of the *H*5 Tyr122 and two F···HO interactions between its Fs and the Tyr122 OH are observed. One indole NH could H-bond with the *H*5 Arg119 C = O and the other indole NH could H-bond with the *H*1 Pro46 C = O. The pose in Figure S6B suggests a H-bond between one F of the cation and the helix *H*12 Phe261 NH and hydrophobic interactions between its phenyl ring and *H*4 Phe112 and Leu113, H11 Leu239 and *H*12 Ile260 side-chains. One indole NH of the cation could H-bond with the *H*5 Glu114 CO_2H or the *H*4 Ser110 OH.

Docking of Csn-B to the apo-NR4A1 LBD (PDB 2QW4)

Our docked pose for Csn-B showed that its 2-octanoyl side chain could extend into the interior of the allosteric pocket to form van der Waals contacts with several pocket residues (Figure 3E). Its 3-OH and 5-OH groups could form H-bonds with the loop L7–8 Val167 backbone C = O and the helix H7 His163 backbone C = O and N, respectively. A partial π–π stacking interaction could occur between the phenyl rings of Csn-B and the H5 Tyr122.

Supplemental synthetic methods and target compound characterizations

General methods

Chemicals and solvents were obtained from commercial sources and used without purification. Unless mentioned, anhydrous and/or oxygen-sensitive reactions were carried out under argon gas. Reactions were monitored by thin-layer chromatography on silica gel (mesh size 60, F_{254}) with visualization under UV light. Unless otherwise specified, the standard work-up involved washing the organic extract with water and brine and then drying it over anhydrous sodium sulfate followed by filtration and concentration under reduced pressure. Chromatography refers to flash column chromatography on silica gel (Merck 60, 230-400 mesh). Most experimental procedures were not optimized. Melting points of compounds were determined in capillary tubes using a Mel-Temp II apparatus and were not corrected. Infrared spectra of powdered or liquid samples were obtained using an FT-IR Mason satellite spectrophotometer. Unless mentioned, ^1H and ^{13}C NMR spectra were obtained on compounds dissolved in $CDCl_3$ or the specified solvent using a 300-MHz Varian Unity Inova or a 400-MHz ECS Jeol spectrometer. Proton chemical shifts are expressed in ppm (δ) relative to $CHCl_3$ as the internal standard. High-resolution mass spectra were determined using an AgilentTechnologies 6224A accurate mass TOF LC/MS system at Sanford-Burnham Medical Research Institute (Lake Nona, FL). A Shimadzu HPLC system was used to analyze the oxidation rate of DIM-Ph-4-CF_3 to DIM-Ph-4-CF_3^+ OMs^- (Supplementary Table 1) and the purity of target molecules (Supplementary Table 2). The purity of compounds used in biological assays was ≥ 95% as determined by HPLC. Compound nomenclature used in these Methods follows that currently employed by *Chemical Abstracts*.

Condensation of indole with 4-substituted benzaldehydes produces the corresponding 4-substituted di(1*H*-indol-3-yl)methylbenzenes (DIM-Ph-4-Xs)

A reported procedure was used to generate the $CeCl_3$•7 H_2O–NaI–SiO_2 catalyst [9]. Briefly, to a solution of $CeCl_3$•7 H_2O (0.9 mmol) and NaI (0.9 mmol) in CH_3CN (21 mL) was added silica gel (13.9 mmol). This mixture was stirred for approximately 21 h before the solvent was removed at reduced pressure to yield a yellow solid. To this solid was added CH_3CN (7.5 mL), indole (6.0 mmol) and the 4-substituted benzaldehyde (3.0 mmol).

The mixture was stirred for 22–52 h, diluted with Et_2O (100 mL) and filtered through a short pad of Celite® (Et_2O rinse). The filtrate was concentrated at reduced pressure, and the residue was purified by chromatography to give the 4-substituted di(1*H*-indol-3-yl)methylbenzene (DIM-Ph-4-X).

3,3′′-[(4-(Trifluoromethyl)phenyl)methylene]bis(1*H*-indole) (DIM-Ph-4-CF_3) [41]

4- (Trifluoromethyl)benzaldehyde (1.05 g, 6.0 mmol) and indole (1.41 g, 12.0 mmol) after reaction for 48 h, work-up and chromatography (22–25% EtOAc/hexane) produced 2.38 g (92%) of DIM-Ph-4-CF_3 as a white solid, mp 86−89° C. IR 3409, 3056, 1456, 1324, 1107, 742 cm^{-1}; ^{1}H NMR δ 5.94 (s, 1H, ArCH), 6.66 (d, *J* = 1.36 Hz, 2H, 2-IndoleH), 6.99–7.04 (m, 2H, 5-IndoleH), 7.16–7.21 (m, 2H, 6-IndoleH), 7.34–7.38 (m, 4H, 4,7-IndoleH), 7.45 (d, *J* = 8.7 Hz, 2H, 2,6-ArH), 7.52 (d, *J* = 8.7 Hz, 2H, 3,5-ArH), 7.95 ppm (bs, 2H, NH). The ^{1}H NMR data are in agreement with those reported [40]; ^{13}C NMR (C^2H_3CN + DMSO-d_6) δ 40.06 (CH), 111.12, 118.76, 119.42, 119.70, 122.16, 123.62, 125.19, 126.80, 128.98, 136.66, 148.11 ppm. The Safe group had synthesized DIM-Ph-4-CF_3 using acetic acid [41] rather than $CeCl_3$•7 H_2O–NaI–SiO_2 as the catalyst.

3,3′′-[(4-Methoxyphenyl)methylene]bis(1*H*-indole) (DIM-Ph-4-OMe) [41]

4-Methoxybenzaldehyde (417 mg, 3.0 mmol) and indole (703 mg, 6.0 mmol) after reaction for 22 h, work-up and chromatography (20–25% EtOAc/hexane) produced 780 mg (74%) of DIM-Ph-4-OMe as a white solid, mp 188–190° C. IR 3410, 3054, 1508, 1455, 1243, 1092, 742 cm^{-1}; ^{1}H NMR δ 3.78 (s, 3H, OCH_3), 5.84 (s, 1H, ArCH), 6.65 (s, 2H, 2-IndoleH), 6.83 (d, *J* = 6.4 Hz, 2H, 3,5-ArH), 7.00 (m, 2H, 5-IndoleH), 7.23 (m, 2H, 6-IndoleH), 7.25 (d, *J* = 6.4 Hz, 2H, 2,6-ArH), 7.35 (d, *J* = 8.2 Hz, 2H, 4-IndoleH), 7.38 (d, *J* = 8.2 Hz, 2H, 7-IndoleH), 7.89 ppm (s, 2H, NH).

Methyl 4-[di(1*H*-indol-3-yl)methyl]benzoate (DIM-Ph-4-CO_2Me) [42]

Methyl 4-formylbenzoate (493 mg, 3.0 mmol) and indole (703 mg, 6.0 mmol) after reaction for 52 h, work-up and chromatography (25–33% EtOAc/hexane) produced 1.02 g (89%) of DIM-Ph-4-CO_2Me as a white solid, mp 99−101° C. IR 3411, 3055, 1706, 1456, 1283, 1110, 741 cm^{-1}; ^{1}H NMR δ 3.77 (s, 3H, OCH_3), 5.89 (s, 1H, ArCH), 6.66 (d, *J* = 2.3 Hz, 2H, 2-IndoleH), 6.82 (t, *J* = 7.3 Hz, 2H, 5-IndoleH), 7.00 (t, *J* = 7.3 Hz, 2H, 6-IndoleH), 7.22 (d, *J* = 7.8 Hz, 2H, 4-IndoleH), 7.30 (d, *J* = 7.8 Hz, 2H, 7-IndoleH), 7.45 (d, *J* = 8.3 Hz, 2H, 2,6-ArH), 7.83 (d, *J* = 8.3 Hz, 2H, 3,5-ArH), 10.83 ppm (s, 2H, NH).

3,3′′-[(4-Chlorophenyl)methylene]bis(1*H*-indole) (DIM-Ph-4-Cl) [42]

4-Chlorobenzaldehyde (435 mg, 3.0 mmol) and indole (703 mg, 6.0 mmol) after reaction for 29 h, work-up and chromatography (20–25% EtOAc/hexane) produced 1.03 g (96%) of DIM-Ph-4-CCl as a white solid, mp 83−86° C. IR 3412, 3054, 1487, 1337, 1089, 742 cm^{-1}; ^{1}H NMR δ 5.81 (s, 1H, ArCH), 6.78 (d, *J* = 2.3 Hz, 2H, 2-IndoleH), 6.83 (t, *J* = 7.32 Hz, 2H, 5-IndoleH), 7.00 (t, *J* = 7.32 Hz, 2H, 6-IndoleH), 7.22 (d, *J* = 7.8 Hz, 2H, 4-IndoleH), 7.26–7.33 (m, 6H, 7-IndoleH, 2,6-ArH, 3,5-ArH), 10.80 ppm (s, 2H, NH).

4-[Di(1*H*-indol-3-yl)methyl]benzoic acid (DIM-Ph-4-CO_2H) [42]

To a stirred solution of DIM-Ph-4-CO_2Me (700 mg, 1.84 mmol) in MeOH (20 mL) was added 5 M aq NaOH (1.84 mL, 9.20 mmol). This mixture was heated at reflux under argon for 50 min, cooled to room temperature and acidified (1 N HCl, 40 mL) and extracted (EtOAc, 130 mL). The organic extract was washed (brine) and dried. The residue obtained on concentration was washed (pentane and CH_2Cl_2) to afford 590 mg (88%) of DIM-Ph-4-CO_2H as a white solid, mp 249–250° C. IR 3410, 3051, 1697, 1454, 1330, 742 cm^{-1}; ^{1}H NMR (CD_3OD) δ 5.92 (s, 1H, ArCH), 6.65 (d, *J* = 2.3 Hz, 2H, 2-IndoleH), 6.87 (t, *J* = 7.5 Hz, 2H, 5-IndoleH), 7.04 (t, *J* = 7.5 Hz, 2H, 6-IndoleH), 7.25 (d, *J* = 8.2 Hz, 2H, 4-IndoleH), 7.32 (d, *J* = 8.2 Hz, 2H, 7-IndoleH), 7.43 (d, *J* = 8.3 Hz, 2H, 2,6-ArH), 7.92 ppm (d, *J* = 8.3 Hz, 2H, 3,5-ArH).

Oxidation of di(1*H*-indol-3-yl)methyl(4-X-benzene)s to their di(1*H*-indol-3-yl)(4-X-phenyl)methylium mesylates

A reported procedure for the synthesis of tris(1-alkylindol-3-yl)methylium salts [4] was adapted. To a solution of the di(1*H*-indol-3-yl)(4-X-phenyl)methane (0.5 mmol) in 1-butanol (5 mL) was added activated carbon powder (Norit®, 10–25 mg) and MsOH (1.5 mmol). This mixture was stirred for more than 20 h under air and then filtered through a short pad of Celite®. The filtrate was washed (water) and concentrated under reduced pressure. The brown residue was triturated (Et_2O), collected by filtration using a sintered glass filter (Et_2O wash) and dried at reduced pressure. The resultant solid was washed (Et_2O, 4Í) and dried to give the product as a reddish-brown powder. Replacing 1-butanol by 2-butanol gave a similar yield.

Di(1*H*-indol-3-yl)(4-trifluoromethylphenyl) methylium mesylate

(DIM-Ph-4-$CF_3^+$$OMs^-$)

Procedure A. DIM-Ph-4-CF_3 (195 mg, 0.5 mmol), MsOH (97 μL, 1.5 mmol) and activated carbon powder (10 mg, 0.8 mmol) in 1-butanol after reaction for 21 h, work-up and purification produced 38 mg (16%) of DIM-Ph-4-CF_3^+ OMs^- as a reddish-brown powder, mp 245−248° C (dec). IR 3115, 2943, 1488, 1413, 1178, 1120, 757 cm^{-1}; ^1H NMR (DMSO-d_6) δ 2.30 (s, 3H, CH_3SO_3), 6.66 (bs, 2H, 7-IndoleH), 7.18 (t, *J* = 6.8 Hz, 2H, 5-IndoleH), 7.40 (t, *J* = 6.8 Hz, 2H, 6-IndoleH), 7.70 (d, *J* = 7.4 Hz, 2H, 4-IndoleH), 7.89 (d, *J* = 6.8 Hz, 2H, 2,6-ArH), 8.05 (d, *J* = 6.8Hz, 2H, 3,5-ArH), 8.68 ppm (bs, 2H, 2-IndoleH); ^{13}C NMR (C^2H_3CN + DMSO-d_6) δ 40.01 (CH_3 in OMs^-), 115.67, 122.58, 123.05, 123.60, 125.56, 126.31, 126.95, 127.09, 127.43, 134.18, 141.07, 148.47, 168.55 (C^+) ppm. HRMS calcd $C_{24}H_{16}F_3N_2$ $[M + H]^+$ 389.1260, found 389.1236.

Procedure B. Pure O_2 was bubbled for 46 h into a stirred mixture of DIM-Ph-4-CF_3 (49 mg, 0.125 mmol), MsOH (24 μL, 0.375 mmol) and activated carbon powder (8 mg, 0.6 mmol) in 1-butanol. The same work-up and purification as in Procedure A produced 22 mg (36%) of DIM-Ph-4-CF_3^+ OMs^- as a reddish-brown powder. Bubbling O_2 into the mixture for 117 h produced 36 mg (60%).

Di(1*H*-indol-3-yl)(4-methoxycarbonylphenyl) methylium mesylate

(DIM-Ph-4-$CO_2Me^+$$OMs^-$)

Procedure A. DIM-Ph-4-CO_2Me (190 mg, 0.5 mmol) and activated carbon powder (25 mg, 2.1 mmol) in 1-butanol after reaction for 43 h under air, work-up and purification produced 31 mg (13%) of DIM-Ph-4-CO_2Me^+ OMs^- as a brown powder, mp 237−240° C (dec). IR 3118, 2891, 1732, 1488, 1416, 1197, 1122, 752 cm^{-1}; ^1H NMR (CD_3OD) δ 2.68 (s, 3H, CH_3SO_3), 4.01 (s, 3H, OCH_3), 6.83 (d, *J* = 6.9 Hz, 2H, 7-IndoleH), 7.16 (t, *J* = 7.3 Hz, 2H, 5-IndoleH), 7.42 (t, *J* = 7.3 Hz, 2H, 6-IndoleH), 7.68 (d, *J* = 8.2 Hz, 2H, 4-IndoleH), 7.79 (d, *J* = 8.2 Hz, 2H, 2,6-ArH), 8.29 (d, *J* = 8.2 Hz, 2H, 3,5-ArH), 8.38 ppm (bs, 2H, 2-IndoleH). HRMS calcd $C_{25}H_{19}N_2O_2$ $[M + H]^+$ 379.1441, found 379.1416.

Procedure B. Pure O_2 was bubbled for 46 h into a stirred mixture of DIM-Ph-4-CO_2Me (48 mg, 0.125 mmol), MsOH (24 μL, 0.375 mmol) and activated carbon powder (8 mg, 0.6 mmol) in 1-butanol. The same work-up and purification as in Procedure A produced 19 mg (32%) of DIM-Ph-4-CO_2Me^+ OMs^- as a reddish-brown powder.

Di(1*H*-indol-3-yl)(4-chlorophenyl)methylium mesylate (DIM-Ph-4-Cl^+ OMs^-)

DIM-Ph-4-Cl (357 mg, 1.0 mmol) and activated carbon powder (50 mg, 4.2 mmol) in 1-butanol after reaction for 45 h under air, work-up and purification produced 123 mg (27%) of DIM-Ph-4-CCl^+ OMs^- as a brown powder, mp 193−196° C (dec). IR 3108, 2883, 1484, 1412, 1175, 1117, 751 cm^{-1}; ^1H NMR (CD_3OD) δ 2.69 (s, 3H, CH_3SO_3), 6.90 (d, *J* = 8.2 Hz, 2H, 7-IndoleH), 7.16 (t, *J* = 7.3 Hz, 2H, 5-IndoleH), 7.42 (t, *J* = 7.3 Hz, 2H, 6-IndoleH), 7.65−7.72 (m, 6H, 4-IndoleH, 2,6-ArH, 3,5-ArH), 8.31 ppm (bs, 2H, 2-IndoleH). HRMS calcd $C_{23}H_{16}ClN_2$ $[M + H]^+$ 355.0997, found 355.0969.

Di(1*H*-indol-3-yl)(4-methoxyphenyl)methylium mesylate (DIM-Ph-4-OMe^+ OMs^-)

DIM-Ph-4-OMe (264 mg, 0.75 mmol) and activated carbon powder (40 mg, 3.3 mmol) in 1-butanol after reaction for 30 h under air, work-up and purification produced 97 mg (27%) of DIM-Ph-4-OMe^+ OMs^- as a brown powder, mp 247−250° C (dec). IR 3109, 2933, 1483, 1412, 1171, 1123, 746 cm^{-1}; ^1H NMR (CD_3OD) δ 2.63 (s, 3H, CH_3SO_3), 3.96 (s, 3H, OCH_3), 6.90 (d, *J* = 7.8 Hz, 2H, 7-IndoleH), 7.01 (t, *J* = 7.3 Hz, 2H, 5-IndoleH), 7.16 (d, *J* = 7.8 Hz, 2H, 3,5-ArH), 7.26 (t, *J* = 7.3 Hz, 2H, 6-IndoleH), 7.49 (d, *J* = 7.8 Hz, 2H, 4-IndoleH), 7.59 (d, *J* = 7.8 Hz, 2H, 2,6-ArH), 7.95 ppm (bs, 2H, 2-IndoleH). HRMS calcd $C_{24}H_{19}N_2O$ $[M + H]^+$ 351.1492, found 351.1467.

Di(1*H*-indol-3-yl)(4-carboxyphenyl)methylium mesylate (DIM-Ph-4-CO_2H^+ OMs^-)

DIM-Ph-4-CO_2H (275 mg, 0.75 mmol) and activated charcoal (40 mg, 3.3 mmol) in 1-butanol after reaction for 120 h under air, work-up and purification produced 22 mg (6%) of DIM-Ph-4-CO_2H^+ OMs^- as a brown powder mp >260° C (dec). IR 2960, 2866, 1484, 1413, 1174, 1124, 1054, 668 cm^{-1}; ^1H NMR (CD_3OD) δ 2.69 (s, 3H, CH_3SO_3), 6.84 (bs, 2H, 7-IndoleH), 7.18 (t, *J* = 7.8 Hz, 2H, 5-IndoleH), 7.42 (t, *J* = 7.8 Hz, 2H, 6-IndoleH), 7.68 (d, *J* = 8.2 Hz, 2H, 4-IndoleH), 7.78 (d, *J* = 8.2 Hz, 2H, 2,6-ArH), 8.28 (d, *J* = 8.2 Hz, 2H, 3,5-ArH), 8.39 ppm (bs, 2H, 2-IndoleH). HRMS calcd $C_{24}H_{17}N_2O_2$ $[M + H]^+$ 365.1285, found 365.1259.

Oxidation of 4-Substituted Di(1*H*-indol-3-yl) methylbenzenes to

(*E*)-3-[(1*H*-indol-3-yl)(4-X-phenyl)methylene]-3*H*-indoles (X = CF_3 and CO_2Me)

A reported method [6] was used. To a stirred solution of the DIM-Ph-4-X in MeCN was added 2,3-dichloro-

5,6-dicyano-p-benzoquinone (DDQ) and stirring was continued at room temperature for 2–3 h. Chromatography produced a dark-red solid, which was washed with EtOAc ($X = CF_3$) or MeCN ($X = CO_2Me$) and then with ether. The crude product was purified by chromatography to give the I(Ph-4-X)MI.

(*E*)-3-((1*H*-indol-3-yl)(4-trifluoromethylphenyl) methylene)-3*H*-indole (I(Ph-4-CF_3)MI)

DIM-Ph-4-CF_3 (195 mg, 0.5 mmol) and DDQ (136 mg, 0.6 mmol) after reaction for 2 h 10 min, work-up and chromatography (5–10% MeOH/CH_2Cl) produced 147 mg (76%) of I(Ph-4-CF_3)MI as an orange powder, mp 241−244° C (dec). IR ($CHCl_3$) 3084, 2914, 2808, 1548, 1475, 1415, 1322, 1133, 1103, 1064, 739 cm^{-1}; ^1H NMR (DMSO-d_6) δ 6.58 (bs, 2H, 7-IndoleH), 6.94 (t, *J* = 7.3 Hz, 2H, 5-IndoleH), 7.20 (t, *J* = 7.3 Hz, 2H, 6-IndoleH), 7.54 (d, *J* = 7.8 Hz, 2H, 4-IndoleH), 7.69 (bs, 2H, 2,6-ArH), 7.91 (d, *J* = 7.8 Hz, 2H, 3,5-ArH), 8.02 ppm (bs, 2H, 2-IndoleH); ^{13}C NMR (C^2H_3CN + DMSO-d_6) δ 112.38, 116.24, 119.42, 122.32, 123.80, 124.76, 125.07, 126.69, 128.01, 128.47, 133.64, 143.84, 148.33 ppm. HRMS calcd $C_{24}H_{15}F_3N_2$ $[M + H]^+$ 389.1266, found 389.1258.

(*E*)-3-((1*H*-indol-3-yl)(4-methoxycarbonylphenyl) methylene)-3*H*-indole

(I(Ph-4-CO_2Me)MI)

DIM-Ph-4-CO_2Me (230 mg, 0.60 mmol) and DDQ (165 mg, 0.73 mmol) after reaction for 3 h, work-up and chromatography (7–10% MeOH/CH_2Cl_2) produced 173 mg (75%) of I(Ph-4-CO_2Me)MI as a reddish-orange powder, mp 235−238° C (dec). IR ($CHCl_3$) 3103, 2949, 2812, 1723, 1566, 1485, 1419, 1279, 1176, 1105, 747 cm^{-1}; ^1H NMR (DMSO-d_6) δ 3.91 (s, 3H, OCH_3), 6.61 (bs, 2H, 7-IndoleH), 6.91 (t, *J* = 7.3 Hz, 2H, 5-IndoleH), 7.18 (t, *J* = 7.3 Hz, 2H, 6-IndoleH), 7.52 (d, *J* = 7.8 Hz, 2H, 4-IndoleH), 7.60 (d, *J* = 8.2 Hz, 2H, 2,6-ArH), 8.08 (bs, 2H, 2-IndoleH), 8.10 ppm (d, *J* = 8.2 Hz, 2H, 3,5-ArH). HRMS calcd $C_{25}H_{18}N_2O_2$ $[M + H]^+$ 379.1447, found 379.1440.

Conversion of (*E*)-3-((1*H*-indol-3-yl)(4-X-phenyl) methylene)-3*H*-indoles into their di(1*H*-indol-3-yl)(4-X-phenyl)methylium mesylates (X = CF_3 and CO_2Me)

To a solution of I(Ph-4-X)MIs (1 equiv.) in 1-butanol (5.0 mL) was added MsOH (8 equiv.). This solution was stirred for approx. 2–3 h at room temperature and then washed (H_2O). The solvents were removed at reduced pressure to give after drying the mesylates DIM-Ph-4-X^+ OMs^-s.

Di(1*H*-indol-3-yl)(4-trifluoromethylphenyl) methylium mesylate

(DIM-Ph-4-$CF_3^+$$OMs^-$)

I(Ph-4-CF_3)MI (12 mg, 0.03 mmol) and MsOH (16 μL, 0.24 mmol) after reaction for 2 h 35 min and work-up produced 10 mg (68%) of DIM-Ph-4-CF_3^+ OMs^- as a reddish-brown powder. IR ($CHCl_3$) 1169 cm^{-1} (broad, S = O stretch). The ^1H NMR spectrum (DMSO-d_6) was identical to that obtained by the oxidation of Ph-4-CF_3 using O_2/activated C/MsOH.

Di(1*H*-indol-3-yl)(4-methoxycarbonylphenyl) methylium mesylate

(DIM-Ph-4-$CO_2Me^+$$OMs^-$)

I(Ph-4-CO_2Me)MI (23 mg, 0.06 mmol) and MsOH (32 μL, 0.48 mmol) after reaction for 3 h 10 min and work-up produced 19 mg (65%) of DIM-Ph-4-CO_2Me^+ OMs^- as a reddish-brown powder. IR ($CHCl_3$) 1171 cm^{-1} (broad, S = O stretch). The ^1H NMR spectrum of this product (DMSO-d_6) was identical to that obtained by the oxidation of Ph-4-CO_2Me using the O_2/activated carbon/ MsOH method.

Differential scanning calorimetry

Experiments were performed at a scanning rate of 1 K/min under 3.0 atm of pressure using an N-DSC II differential scanning calorimeter (Calorimetry Sciences Corp.). Samples contained NR4A1 LBD (20 μM, 0.51 mg/ml) alone or with compound (30 or 60 μM), and 5% DMSO in PBS, pH 7.4. The reference contained 5% DMSO in PBS. Data were analyzed using the NanoAnalyse software package (TA Instruments). Melting temperature (T_m) corresponds to the maximum thermal transition temperature. Calorimetric enthalpy (ΔH) was calculated as the area under the excess heat capacity function (C_p).

Computational methods

Volume calculation

Chemical group volumes (CO_2Me, 53.6 Å^3; CF_3, 40.3 Å^3; CO_2H, 36.0 Å^3; OMe, 34.6 Å^3; and Cl, 22.6 Å^3) were calculated using the free online service for calculating molecular properties provided by Molinspiration Cheminformatics (Nova ulica, Slovensky Grob, Slovak Republic) [7].

Molecular modeling

Docking of the cation (DIM-Ph-4-CF_3^+) of DIM-Ph-4-CF_3^+ OMs^- into the pocket corresponding to allosteric

site 1 in the crystal structure of the human NR4A1 (TR3) LBD protein (PDB 2QW4) [43] employed BioMed Cache vs. 6.2 software, which is no longer supported by Fujitsu Limited, and our previously described methods [44]. Briefly, the LBP was derived by selecting all neighboring residues within a 10-Å radius to ensure that all residues were encompassed. In the docking process, the energy-minimized pose of Csn-B was kept rigid to maintain the geometry used for docking by Wu and colleagues [45], whereas the carbocation was allowed to be flexible. In both cases, the side chains of pocket residues were allowed to be flexible. The docked poses for Csn-B and DIM-Ph-4-CF_3^+ (Figure 3E) were analyzed by measuring inter-atom distances after superposing the helical backbones of the docked NR4A1 LBD poses.

Docking of DIM-Ph-4-CF_3^+ into allosteric sites 1 and 2 on the structure (PDB 3V3Q) [11] of the complex of the NR4A1 LBD with two molecules of the Csn-B analogue (structure in Figure S1) used the GOLD docking engine in the Discovery Studio 3.5 package (Accelrys Inc.). The LBP for each site was derived by selecting all neighboring residues within an 8-Å radius of the analogue [11]. The geometry of DIM-Ph-4-CF_3^+ was optimized before docking and during docking was allowed to be flexible, whereas the side chains of pocket residues were kept rigid. Possible interactions between DIM-Ph-4-CF_3^+ and the residues lining both sites were analyzed and are shown in Figure S6.

Cancer cell line characteristics

HCT-116 colorectal carcinoma cells are poorly differentiated, express a constitutively active mutant p21ras (Ki-*ras*) proto-oncogene [13] and a mutant β-catenin, which is not inhibited by wild-type APC [14], and over-express c-*myc* [13]. This cell line also expresses wild-type p53, PPARγ and NR4A1 [12]. Of the breast cancer lines, MCF- 7 expresses estrogen receptors and its growth is estrogen-dependent [15, 18], whereas MDA-MB-231 does not express estrogen receptors and its growth is estrogen-independent [18]. Wild-type tumor suppressor p53 and mutant nonfunctional p53 are expressed in MCF-7 and MDA-MB-231 cells, respectively [26]. NR4A1 is expressed in MCF-7 cells [1], but not in MDA-MB-231 cells [1], and PPARγ is expressed in both lines [17, 18]. Of the prostate cancer cell lines, LNCaP cells are androgen-dependent and express the mutant androgen receptor (AR) (Thr877Ala), which is activated by both the androgen dihydrotestosterone (DHT) and the anti-androgen hydroxyflutamide [25]. They also express low levels of PPARγ1 [24] and after treatment with the PPARγ ligand 15d-Pg J_2 express modest levels of PPARγ2 [24]. The calcium ionophore A23187, anti-cancer drug etoposide and androgen induce the expression of NR4A1 [27]. LAPC-4 cells are androgen-dependent and express wild-type AR [23, 28]. 22Rv1 cells are reported to grow independently of androgen in vivo and to express both a DHT and hydroxyflutamide-activated full-length AR(His874Tyr) mutant [25] and a truncated 80-KDa AR mutant [29]. They also express NR4A1 [24], but not PPARγ [24]. The expression of NR4A1 and PPARγ in LAPC-4 cells [23] has not been reported. The growth of PC-3 cells, which are AR null, is androgen independent [25]. They weakly express PPARγ1 [25] and after treatment with 15d-Pg J_2 robustly express PPARγ2 [25], A23187 or etoposide treatment induces their expression of NR4A1 [27]. PC-3 cells do not express p53 [26], whereas the other three lines do [26]. The phosphatase and tensin homologue deleted on chromosome 10 (PTEN) enzyme is reported to antagonize AR transactivation and Akt–PI3K signaling, and its loss is associated with advanced prostate cancer [28]. PTEN is expressed and functional in LACP-4 [28] and 22Rv1 [28, 29] cells, although in the former its expression is reported to be low [29], PTEN is absent in LNCaP and PC-3 cells [28].

REFERENCES

1. Soule HD, Vazguez J, Long A, Albert S, Brennan M. A human cell line from a pleural effusion derived from a breast carcinoma. J Natl Cancer Inst. 1973; 51:1409–16.
2. Contractor R, Samudio IJ, Estrov Z, Harris D, McCubrey JA, Safe SH, Andreeff M, Konopleva, M. A novel ring-substituted diindolylmethane,1,1-bis[3’-(5-methoxyindolyl)]-1-(p-t-butylphenyl) methane, inhibits extracellular signal-regulated kinase activation and induces apoptosis in acute myelogenous leukemia. Cancer Res. 2005; 65:2890–8.
3. Horwitz KB, McGuire WL. Estrogen control of progesterone receptor in human breast cancer. Correlation with nuclear processing of estrogen receptor. J Biol Chem. 1978; 253:2223–8.
4. Lavrenov SN, Luzikov YN, Bykov EE, Reznikova MI, Stepanova EV, Glazunova VA, Volodina YL, Tatarsky VV Jr, Shtil AA, Preobrazhenskaya MN. Synthesis and cytotoxic potency of novel tris(1-alkylindol-3-yl)methylium salts: role of N-alkyl substituents. Bioorg Med Chem. 2010; 18:6905–13.
5. Cailleau R, Young R, Olive M, Reeves WJ Jr. Breast tumor cell lines from pleural effusions. J Natl Cancer Inst. 1974; 53:661–74.
6. He X, Hu S, Liu K, Guo Y, Xu J, Shao S. Oxidized bis(indolyl)methane: a simple and efficient chromogenic-sensing molecule based on the proton transfer signaling mode. Org Lett. 2006; 8:333–6.
7. Molinspiration Cheminformatics. Calculation of molecular properties and bioactivity score. http://www.molinspiration.com/cgi-bin/properties (accessed June 16, 2013).
8. Wu Q, Dawson MI, Zheng Y, Hobbs PD, Agadir A, Jong L, Li Y, Liu R, Lin B, Zhang XK. Inhibition of trans-retinoic acid-resistant human breast cancer cell growth by

retinoid X receptor-selective retinoids. Mol Cell Biol. 1997; 17:6598–608.

9. Bartoli G, Bosco M, Foglia G, Giuliani A, Marcantoni E, Sambri L. Solvent-free indoles addition to carbonyl compounds promoted by $CeCl_3.7H_2O$-NaI-SiO_2: an efficient method for the synthesis of streptindole. Synthesis. 2004; 6:895–900.
10. Kaczmarek L, Zagrodzki B, Kamienski B, Pietrzak M, Schilf W, Les A. Synthesis and NMR study of new derivatives of [2,2'-bipyridyl]-3,3'-diol and [2,2'-bipyridyl] -3-ol. J Mol Struct. 2000; 553:61–72.
11. Liu JJ, Zeng HN, Zhang LR, Zhan YY, Chen Y, Wang Y, Wang J, Xiang SH, Liu WJ, Wang WJ, Chen HZ Shen YM, Su WJ, Huang PQ, et al. A unique pharmacophore for activation of the nuclear orphan receptor Nur77 in vivo and in vitro. Cancer Res. 2010; 70:3628–37.
12. Holla VR, Mann JR, Shi Q, DuBois RN. Prostaglandin E_2 regulates the nuclear receptor NR4A2 in colorectal cancer. J Biol Chem. 2006; 281:2676–82.
13. Katayose D, Gudas J, Nguyen H, Srivastava S, Cowan KH, Seth P. Cytotoxic effects of adenovirus-mediated wild-type p53 protein expression in normal and tumor mammary epithelial cells. Clin Cancer Res. 1995; 1:889–97.
14. Montemurro L, Tonelli R, Fazzina R, Martino V, Marino F, Pession A. Identification of two MLL-MLLT3 (alias MLL-AF9) chimeric transcripts in the MOLM-13 cell line. Cancer Genet Cytogenet. 2004; 154:96–7.
15. Alexopoulou AN, Leao M, Caballero OL, Da Silva L, Reid L, Lakhani SR, Simpson AJ, Marshall JF, Neville AM, Jat PS. Dissecting the transcriptional networks underlying breast cancer: NR4A1 reduces the migration of normal and breast cancer cell lines. Breast Cancer Res. 2010; 12:R51.
16. Hansch C, Leo A, Taft RW. A survey of Hammett substituent constants and resonance and field parameters. Chem. Rev. 1991;91:165–95.
17. Cheng H, Meng J, Wang G, Meng Y, Li Y, Wei D, Fu C, Deng K, Shen A, Wang H, Dai S. Skp2 regulates subcellular localization of PPARg by MEK signaling pathways in human breast cancer. Int J Mol Sci. 2013; 14:16554 69.
18. Malaviya A, Sylvester PW. Mechanisms mediating the effects of g-tocotrienol when used in combination with PPARg agonists or antagonists on MCF-7 and MDA-MB-231 breast cancer cells. Int J Breast Cancer. 2013; 2013:101705.
19. Chhipa RR, Halim D, Cheng J, Zhang HY, Mohler JLIp C, Wu Y. The direct inhibitory effect of dutasteride or finasteride on androgen receptor activity is cell line specific. Prostate. 2013; 73:1483–94.
20. Xia Z, Correa RG, Das JKFarhana L, Castro DJ, Yu J, Oshima RG, Fontana JA, Reed JC, Dawson MI. Analogues of orphan nuclear receptor small heterodimer partner ligand and apoptosis inducer (*E*)-4-[3-(1-adamantyl)-4-hydroxyphenyl]-3-chlorocinnamic acid. 2. Impact of 3-chloro group replacement on inhibition of proliferation and induction of apoptosis of leukemia and cancer cell lines. J Med Chem. 2012; 55:233–49.
21. Kon A, Shih LY, Minamino M, Sanada M, Shiraishi Y, Nagata Y, Yoshida K, Okuno Y, Bando M, Nakato R, Ishikawa S, Sato-Otsubo A, Nagae G, et al. Recurrent mutations in multiple components of the cohesin complex in myeloid neoplasms. Nat Genet. 2013; 45:1232–7.
22. Uemura H, Chang C. Antisense TR3 orphan receptor can increase prostate cancer cell viability with etoposide treatment. Endocrinology. 1998; 139:2329–34.
23. Wang MH, Abreu-Delgado Y, Young CY. Effects of vitamin C on androgen receptor mediated actions in human prostate adenocarcinoma cell line LAPC-4. Urology. 2003; 62:167–71.
24. Marcias G, Erdmann E, Lapouge G, Siebert C, Barthelemy P, Duclos B, Bergerat JP, Ceraline J, Kurtz JE. Identification of novel truncated androgen receptor (AR) mutants including unreported pre-mRNA splicing variants in the 22Rv1 hormone-refractory prostate cancer (PCa) cell line. Hum Mutat. 2010; 31:74–80.
25. Tilley WD, Wilson CM, Marcelli M, McPhaul MJ. Androgen receptor gene expression in human prostate carcinoma cell lines. Cancer Res. 1990; 50:5382–6.
26. Hsieh T, Wu J. Differential expression and regulation of p53 in human prostatic cells. Int J Oncol. 1997; 10:1109–12.
27. Gravina GL, Marampon F, Giusti I. Carosa E, Di Sante S, Ricevuto E, Dolo V, Tombolini V, Jannini EA, Festuccia C. Differential effects of PXD101 (belinostat) on androgen-dependent and androgen-independent prostate cancer models. Int J Oncol. 2012; 40:711–20.
28. Nan B, Snabboon T, Unni E, Yuan XJ, Whang YE, Marcelli M. The PTEN tumor suppressor is a negative modulator of androgen receptor transcriptional activity. J Mol Endocrinol. 2003; 31:169–83.
29. Fraser M, Zhao H, Luoto KR, Lundin C, Coackley C, Chan N, Joshua AM, Bismar TA, Evans A. Helleday T, Bristow RG. PTEN deletion in prostate cancer cells does not associate with loss of RAD51 function: implications for radiotherapy and chemotherapy. Clin Cancer Res. 2012; 18:1015–27.
30. Colmenarejo G. In silico prediction of drug-binding strengths to human serum albumin. Med Res Rev. 2003; 23:275–301.
31. Ghuman J, Zunszain PA, Petitpas I, Bhattacharya AA, Otagiri M, Curry S. Structural basis of the drug-binding specificity of human serum albumin. J Mol Biol. 2005; 353:38–52.
32. Sovolyova N, Healy S, Samali A, Logue SE. Stressed to death - mechanisms of ER stress-induced cell death. Biol Chem. 2013; 395:1–13.
33. Hetz C, Martinon F, Rodriguez D, Glimcher LH. The unfolded protein response: integrating stress signals

through the stress sensor IRE1alpha. Physiol Rev. 2011; 91:1219–43.

34. Woehlbier U, Hetz C. Modulating stress responses by the UPRosome: a matter of life and death. Trends Biochem Sci. 2011; 36:329–37.

35. Liu B, Wu JF, Zhan YY, Chen HZ, Zhang XY, Wu Q. Regulation of the orphan receptor TR3 nuclear functions by c-Jun N terminal kinase phosphorylation. Endocrinology. 2007; 148:34–44.

36. Kuang X, Hu W, Yan M, Wong PK. Phenylbutyric acid suppresses protein accumulation-mediated ER stress in retrovirus-infected astrocytes and delays onset of paralysis in infected mice. Neurochem Int. 2010; 57:738–48.

37. Davenport EL, Moore HE, Dunlop AS, Sharp SY, Workman P, Morgan GJ, Davies FE. Heat shock protein inhibition is associated with activation of the unfolded protein response pathway in myeloma plasma cells. Blood. 2007; 110:2641–9.

38. Zhang XK. Vitamin A and apoptosis in prostate cancer. Endocr Relat Cancer. 2002; 9:87–102.

39. Cao X, Liu W, Lin F, Li H, Kolluri SK, Lin B, Han YH, Dawson MI, Zhang XK. Retinoid X receptor regulates Nur77/TR3-dependent apoptosis [corrected] by modulating its nuclear export and mitochondrial targeting. Mol Cell Biol. 2004; 24:9705–25.

40. Dawson MI, Ye M, Cao X, Farhana L, Hu QY, Zhao Y, Xu LP, Kiselyuk A, Correa RG, Yang L, Hou T, Reed JC, Itkin-Ansari P, et al. Derivation of a retinoid X receptor scaffold from peroxisome proliferator-activated receptor g ligand 1-di(1*H*-indol-3-yl)methyl-4-trifluoromethylbenzene. Chem Med Chem. 2009; 4:1106–1119.

41. Qin C, Morrow D, Stewart J, Spencer K, Porter W, Smith R III, Phillips T, Abdelrahim M, Samudio I, Safe S. A new class of peroxisome proliferator-activated receptor g (PPARg) agonists that inhibit growth of breast cancer cells: 1,1-Bis(3¢-indolyl)-1-(*p*-substituted phenyl)methanes. Mol. Cancer Ther. 2004; 3:247–260.

42. Tjalkens RB and Safe S. Use of diindolylmethane (DIM) compounds and derivatives as neuroprotective agents. Patent US8580843(B2), 2013.

43. Flaig R, Greschik H, Peluso-Iltis C, Moras D. Structural basis for the cell-specific activities of the NGFI-B and the Nurr1 ligand-binding domain. J Biol Chem. 2005; 280:19250–19258.

44. Xia Z, Cao X, Rico-Bautista E, Yu J, Chen L, Chen J, Bobkov A, Wolf DA, Zhang XK, Dawson MI. Relative impact of 3- and 5-hydroxyl groups of cytosporone B on cancer cell viability. Med Chem Commun. 2013; 4:332–339.

45. Zhan Y, Du X, Chen H, Liu J, Zhao B, Huang D, Li G, Xu Q, Zhang M, Weimer BC, Chen D, Cheng Z, Zhang L, et al. Cytosporone B is an agonist for nuclear orphan receptor Nur77. Nat Chem Biol. 2008; 4:548–556.
