# Supplementary material for: Oxidized analogs of Di(1H-indol-3-yl)methyl-4-substituted benzenes are NR4A1-dependent UPR inducers with potent and safe anti-cancer activity
Source: Oncotarget. 2018 May 18;9(38):25057–74. doi: 10.18632/oncotarget.25285 (PMC5982742; doi:10.18632/oncotarget.25285)
Supplement: Supplementary file 2 [file oncotarget-09-25057-s002.docx]

| **Table S3. Characteristics of leukemia cell lines—KG-1, MOLM-13, OCI-AML-2, OCI-AML-3 and THP-1 AML, K562 CML and MOLT-4 T-ALL*^a,b^*** | | | | |
| --- | --- | --- | --- | --- |
| **Cell line** | **Type** | **p53 status** | **Mutations** | **Expression change** |
| KG-1 | AML FAB M0^c,^ [1]  Immature/minimally differentiated myeloblastic | Mutant inactive [3] | 5qNCA [12]  del(5q) [13]  Mxi1 missense [14]  NPM–MLF1 fusion [15]  N-ras oncogene [16]  OGG1(R209Q) [17]  OGG1(R229Q) [18]  PTEN mutant and wt PTEN [19] | c-TRK [33]  SAG (↑) [34]  TNFR p55 and p75 (↓) [35]  TSG101 [36] |
| MOLM-13 | AML FAB M5a [1] relapsed | Wild-type [4] | c-CBL E3 ubiquitin ligase [20]  FLT3-ITD + wt FLT3 [21]  MLL–AF9 fusion (two transcripts) [22] | Myc (deregulated) [2]  RAD21 (↓) [37]  STAG2 (↓) [37] |
| OCI-AML-2 | AML FAB M4 [1] myelomonocytic | Wild-type [5] | DNMT3A(R635W) [23] |  |
| OCI-AML-3 | AML FAB M4 [1] | Wild-type [4] | DNMT3l(R882G) [23]  NPM1c [23] |  |
| THP-1 | AML FAB M5 [2] monocytic | Null (codon 1 26-base Δ) [6] | MLL–AF9 fusion [24]  p53 inactive mutant induced on differentiation [25]  PAI-2 truncated (inactive) [26] | P450arom PI.6 (induced) [38, 39] |
| K562 | CML in blast crisis  Erythroleukemia with AML M6 properties | Null [3, 7] | Apaf-1 mRNA defect [27]  BCR–ABL fusion [28]  p14(ARF) null [29]  p15(CDKN2B) null [29]  p16^INK4a^ (CDKN2A) null [29]  N-APase null [30] | c-myc mRNA (↑) [40]  HSP70A mRNA (↑) [41]  HSP90 (↑) [42]  TSG101 [36] |
| MOLT-4 | ALL  T-cell lymphoma | Active variant [8-10] C terminus truncated | ARAF(A451T) missense [31]  RIZ1 null [32] | Tdnt (↑) [43] |

*^a^* NR4A1 expression was reported to be down-regulated in AML [44] and CML [45] lines and patient samples, [44, 45] although it was reported to be restored after treatment of AML cells with an histone deacetylase inhibitor [46]. In some T-LL cell lines the transcription factor (TF) NKX2-5 was ectopically activated leading to the up-regulation and activation of the TF MEF2C, which inhibited NR4A1 expression [47].  *^b^*Abbreviations and definitions: AF9, transcriptional coactivator of MYC that when fused to MLL is constitutively active; ARAF, Raf family member gene that is hypomethylated in smokers but not in nonsmokers; BCR–ABL, breakpoint cluster region–Abelsone kinase fusion that is constitutively active and a cause of CML; c-CBL, casistas B-cell lymphoma E3 ubiquitin ligase that targets tyrosine kinases for degradation; c-TRK, nerve growth factor high-affinity receptor; Δ, deletion; del(5q), interstitial deletion on chromosome 5 at band q31; Dnmt1, deoxynucleotide methyltransferase 1; DNMT3A, DNA methyltransferase 3A; FAB, French-American-British AML cell classification system; FLT3–ITD, FMS-like tyrosine kinase-internal tandem duplication leading to constitutive activation of mTOR and Akt signaling; HSP70A, heat shock protein 70A that when constitutively expressed causes enhanced resistance to thermal killing; MLF1, myelodysplasia/myeloid leukemia factor 1, located in the cytoplasm, except when the t(3;5)(q25.1;q34) fusion with nucleoplosphin causes relocalization to the nucleus; Mxi1, Max interactor protein 1, which antagonizes c-Myc by heterodimerizing with its partner Max on E-box sites; N-APase, N-alkaline phosphatase; NPM, nucleophosphin; OGG1, 8-oxoguanine DNA glycosylase 1, which excises bases from oxidatively damaged DNA; OGG1(R229Q), thermolabile and inactive OGG1 mutant; p14 (ARF), 14-KDa alternate reading frame of INK4a/ARF locus, a tumor suppressor inactivating Mdm2 to release p53; p15 (CDKN2B), 15-KDa cyclin-dependent kinase 6 inhibitor and tumor suppressor; p16 (CDKN2A), 16-KDa cyclin-dependent kinase 4 inhibitor and tumor suppressor; P450arom, P450 aromatase/CYP19 gene, whose expression is phorbol ester-induced from the I.6 promoter; PAI-2, plasminogen activation inhibitor 2, which enhances adhesion and reduces proliferation; PTEN, phosphatase and tensin homolog; RAD21 (SCC1), a cohesin subunit with role in maintaining embryonic stem cell pluripotency; SAG, sensitive to apoptosis gene or RBX2, RING component of SKP2, Sullin and F-box protein E3 ubiquitin ligase degrades redox-induced antioxidant proteins such as c-Jun, HIF-1α, NOXA, p21 and procaspase-3 in a cell-context dependent manner to inhibit apoptosis; STAG2, cohesin subunit SA2, a vertebrate SCC3 homolog with role in sister centromere cohesion; Tdnt, terminal deoxynucleotidyl transferase; TNFR p55, tumor necrosis factor receptor that binds TNF-α to induce NF-κΒ, growth inhibition and necrosis of KG-1 cells; TSG101, tumor susceptibility gene 101; wt, wild-type.

***^b^*References cited in Table S3:**

1. Dijk M, Murphy E, Morrell R, Knapper S, O’Dwyer M, Samali A, Szegezdi E. The proteasome inhibitor bortezomib sensitizes AML with myelomonocytic differentiation to TRAIL mediated apoptosis. Cancers (Basel). 2011; 3:1329–50.

2. Martino V, Tonelli R, Montemurro L, Franzoni M, Marino F, Fazzina R, Pession A. Down-regulation of MLL-AF9, MLL and MYC expression is not obligatory for monocyte-macrophage maturation in AML-M5 cell lines carrying t(9;11)(p22;q23). Oncol Rep. 2006; 15:207–11.

3. Shiohara M, Akashi M, Gombart AF, Yang R, Koeffler HP. Tumor necrosis factor alpha: posttranscriptional stabilization of WAF1 mRNA in p53-deficient human leukemic cells. J Cell Physiol. 1996; 166:568–76.

4. Kojima K, Konopleva M, Samudio IJ, Ruvolo V, Andreeff M. Mitogen-activated protein kinase kinase inhibition enhances nuclear proapoptotic function of p53 in acute myelogenous leukemia cells. Cancer Res. 2007; 67:3210–19.

5. Koistinen P, Zheng A, Säily M, Siitonen T, Mäntymaa P, Savolainen ER. Superior effect of 9-cis retinoic acid (RA) compared with all-trans RA and 13-cis RA on the inhibition of clonogenic cell growth and the induction of apoptosis in OCI/AML-2 subclones: is the p53 pathway involved? Br J Haematol. 2002; 118:401–10.

6. Akashi M, Osawa Y, Koeffler HP, Hachiya M. p21^WAF1^ expression by an activator of protein kinase C is regulated mainly at the post-transcriptional level in cells lacking p53: important role of RNA stabilization. Biochem J. 1999; 337:607–16.

7. Lübbert M, Miller CW, Crawford L, Koeffler HP. p53 in chronic myelogenous leukemia. Study of mechanisms of differential expression. J Exp Med. 1988; 167:873–86.

8. Chow VT, Quek HH, Tock EP. Alternative splicing of the p53 tumor suppressor gene in the Molt-4 T-lymphoblastic leukemia cell line. Cancer Lett. 1993; 73:141–48.

9. O’Connor PM, Jackman J, Bae I, Myers TG, Fan S, Mutoh M, Scudiero DA, Monks A, Sausville EA, Weinstein JN, Friend S, Fornace AJ Jr, Kohn KW. Characterization of the p53 tumor suppressor pathway in cell lines of the National Cancer Institute anticancer drug screen and correlations with the growth-inhibitory potency of 123 anticancer agents. Cancer Res. 1997; 57:4285–300.

10. Szkanderová S, Vávrová J, Rézacová M, Vokurková D, Pavlová S, Smardová J, Stulík J. Gamma irradiation results in phosphorylation of p53 at serine-392 in human T-lymphocyte leukaemia cell line MOLT-4. Folia Biol (Praha). 2003; 49:191–96.

11. Chow V, Ang W. The gene encoding the p53-regulated inhibitor of cdks (pic1) is not expressed in the molt-4 leukemia-cell line with p53 truncated at the carboxyl-terminus, and harbors a nucleotide substitution at codon-31 in several other cancer cell-lines. Int J Oncol. 1995; 6:871–76.

12. Hu Z, Gomes I, Horrigan SK, Kravarusic J, Mar B, Arbieva Z, Chyna B, Fulton N, Edassery S, Raza A, Westbrook CA. A novel nuclear protein, 5qNCA (LOC51780) is a candidate for the myeloid leukemia tumor suppressor gene on chromosome 5 band q31. Oncogene. 2001; 20:6946–54.

13. Xie H, Hu Z, Chyna B, Horrigan SK, Westbrook CA. Human mortalin (HSPA9): a candidate for the myeloid leukemia tumor suppressor gene on 5q31. Leukemia. 2000; 14:2128–34.

14. Guo XL, Pan L, Zhang XJ, Suo XH, Niu ZY, Zhang JY, Wang F, Dong ZR, Da W, Ohno R. Expression and mutation analysis of genes that encode the Myc antagonists Mad1, Mxi1 and Rox in acute leukaemia. Leuk Lymphoma. 2007; 48:1200–07.

15. Yoneda-Kato N, Look AT, Kirstein MN, Valentine MB, Raimondi SC, Cohen KJ, Carroll AJ, Morris SW. The t(3;5)(q25.1;q34) of myelodysplastic syndrome and acute myeloid leukemia produces a novel fusion gene, NPM-MLF1. Oncogene. 1996; 12:265–75.

16. Janssen JW, Steenvoorden AC, Collard JG, Nusse R. Oncogene activation in human myeloid leukemia. Cancer Res. 1985; 45:3262–67.

17. Hyun JW, Choi JY, Zeng HH, Lee YS, Kim HS, Yoon SH, Chung MH. Leukemic cell line, KG-1 has a functional loss of hOGG1 enzyme due to a point mutation and 8-hydroxydeoxyguanosine can kill KG-1. Oncogene. 2000; 19:4476–79.

18. Hill JW, Evans MK. A novel R229Q OGG1 polymorphism results in a thermolabile enzyme that sensitizes KG-1 leukemia cells to DNA damaging agents. Cancer Detect Prev. 2007; 31:237–43.

19. Liu TC, Lin PM, Chang JG, Lee JP, Chen TP, Lin SF. Mutation analysis of PTEN/MMAC1 in acute myeloid leukemia. Am J Hematol. 2000; 63:170–75.

20. Caligiuri MA, Briesewitz R, Yu J, Wang L, Wei M, Arnoczky KJ, Marburger TB, Wen J, Perrotti D, Bloomfield CD, Whitman SP. Novel c-CBL and CBL-b ubiquitin ligase mutations in human acute myeloid leukemia. Blood. 2007; 110:1022–24.

21. Seedhouse C, Grundy M, Shang S, Ronan J, Pimblett H, Russell N, Pallis M. Impaired S-phase arrest in acute myeloid leukemia cells with a FLT3 internal tandem duplication treated with clofarabine. Clin Cancer Res. 2009; 15:7291–98.

22. Montemurro L, Tonelli R, Fazzina R, Martino V, Marino F, Pession A. Identification of two MLL-MLLT3 (alias MLL-AF9) chimeric transcripts in the MOLM-13 cell line. Cancer Genet Cytogenet. 2004; 154:96–97.

23. Tiacci E, Spanhol-Rosseto A, Martelli MP, Pasqualucci L, Quentmeier H, Grossmann V, Drexler HG, Falini B. The NPM1 wild-type OCI-AML2 and the NPM1-mutated OCI-AML3 cell lines carry DNMT3A mutations. Leukemia. 2012; 26:554–57.

24. Odero MD, Zeleznik-Le NJ, Chinwalla V, Rowley JD. Cytogenetic and molecular analysis of the acute monocytic leukemia cell line THP-1 with an MLL-AF9 translocation. Genes Chromosomes Cancer. 2000; 29:333–38.

25. Hsu JW, Huang HC, Chen ST, Wong CH, Juan HF. Ganoderma lucidum polysaccharides induce macrophage-like differentiation in human leukemia THP-1 cells via caspase and p53 activation. Evid Based Complement Alternat Med. 2011; 2011:358717.

26. Katsikis J, Yu H, Maurer F, Medcalf R. The molecular basis for the aberrant production of plasminogen activator inhibitor type 2 in THP-1 monocytes. Thromb Haemost. 2000; 84:468–73.

27. Furukawa Y, Sutheesophon K, Wada T, Nishimura M, Saito Y, Ishii H, Furukawa Y. Methylation silencing of the Apaf-1 gene in acute leukemia. Mol Cancer Res. 2005; 3:325–34.

28. Shtivelman E, Lifshitz B, Gale RP, Roe BA, Canaani E. Alternative splicing of RNAs transcribed from the human abl gene and from the bcr-abl fused gene. Cell. 1986; 47:277–84.

29. Gursky S, Olopade OI, Rowley JD. Identification of a 1.2 Kb cDNA fragment from a region on 9p21 commonly deleted in multiple tumor types. Cancer Genet Cytogenet. 2001; 129:93–101.

30. Klein E, Ben-Bassat H, Neumann H, Ralph P, Zeuthen J, Polliack A, Vánky F. Properties of the K562 cell line, derived from a patient with chronic myeloid leukemia. Int J Cancer. 1976; 18:421–31.

31. Lee JW, Soung YH, Kim SY, Park WS, Nam SW, Min WS, Kim SH, Lee JY, Yoo NJ, Lee SH. Mutational analysis of the ARAF gene in human cancers. APMIS. 2005; 113:54–57.

32. Shimura H, Mori N, Wang YH, Okada M, Motoji T. Aberrant methylation and decreased expression of the RIZ1 gene are frequent in adult acute lymphoblastic leukemia of T-cell phenotype. Leuk Lymphoma. 2012; 53:1599–609.

33. Kaebisch A, Brokt S, Seay U, Lohmeyer J, Jaeger U, Pralle H. Expression of the nerve growth factor receptor c-TRK in human myeloid leukaemia cells. Br J Haematol. 1996; 95:102–09.

34. Tan M, Li Y, Yang R, Xi N, Sun Y. Inactivation of SAG E3 ubiquitin ligase blocks embryonic stem cell differentiation and sensitizes leukemia cells to retinoid acid. PLoS One. 2011; 6:e27726.

35. Sato T, Watanabe N, Yamauchi N, Sasaki H, Kobayashi D, Tsuji N, Okamoto T, Hagino T, Niitsu Y. Differentiation induction by a tumor-necrosis-factor mutant 471 in human myelogenous leukemic cells via tumor-necrosis-factor receptor-p55. Int J Cancer. 1998; 78:223–32.

36. Lin PM, Liu TC, Chang JG, Chen TP, Lin SF. Aberrant TSG101 transcripts in acute myeloid leukaemia. Br J Haematol. 1998; 102:753–58.

37. Kon A, Shih LY, Minamino M, Sanada M, Shiraishi Y, Nagata Y, Yoshida K, Okuno Y, Bando M, Nakato R, Ishikawa S, Sato-Otsubo A, Nagae G, et al. Recurrent mutations in multiple components of the cohesin complex in myeloid neoplasms. Nat Genet. 2013; 45:1232–37.

38. Shozu M, Zhao Y, Simpson ER. Estrogen biosynthesis in THP1 cells is regulated by promoter switching of the aromatase (CYP19) gene. Endocrinology. 1997; 138:5125–35.

39. Shozu M, Zhao Y, Bulun SE, Simpson ER. Multiple splicing events involved in regulation of human aromatase expression by a novel promoter, I.6. Endocrinology. 1998; 139:1610–17.

40. Tonini GP, Radzioch D, Gronberg A, Clayton M, Blasi E, Benetton G, Varesio L. Erythroid differentiation and modulation of c-myc expression induced by antineoplastic drugs in the human leukemic cell line K562. Cancer Res. 1987; 47:4544–47.

41. Mivechi NF, Rossi JJ. Use of polymerase chain reaction to detect the expression of the M_r_ 70,000 heat shock genes in control or heat shock leukemic cells as correlated to their heat response. Cancer Res. 1990; 50:2877–84.

42. Záčková M, Moučková D, Lopotová T, Ondráčková Z, Klamová H, Moravcová J. Hsp90 - a potential prognostic marker in CML. Blood Cells Mol Dis. 2013; 50:184–89.

43. Srivastava BI. Deoxynucleotide-polymerizing enzyme activities in T- and B-cells of acute lymphoblastic leukemia origin. Cancer Res. 1976; 36:1825–30.

44. Mullican SE, Zhang S, Konopleva M, Ruvolo V, Andreeff M, Milbrandt J, Conneely OM. Abrogation of nuclear receptors Nr4a3 and Nr4a1 leads to development of acute myeloid leukemia. Nat Med. 2007; 13:730–35.

45. Ramirez-Herrick AM, Mullican SE, Sheehan AM, Conneely OM. Reduced NR4A gene dosage leads to mixed myelodysplastic/myeloproliferative neoplasms in mice. Blood. 2011; 117:2681–90.

46. Zhou L, Ruvolo VR, McQueen T, Chen W, Samudio IJ, Conneely O, Konopleva M, Andreeff M. HDAC inhibition by SNDX-275 (Entinostat) restores expression of silenced leukemia-associated transcription factors Nur77 and Nor1 and of key pro-apoptotic proteins in AML. Leukemia. 2013; 27:1358–68.

47. Nagel S, Meyer C, Quentmeier H, Kaufmann M, Drexler HG, MacLeod RA. MEF2C is activated by multiple mechanisms in a subset of T-acute lymphoblastic leukemia cell lines. Leukemia. 2008; 22:600–07.
